# Temporal Profiling of Host Proteome against Different *M. tuberculosis* Strains Reveals Delayed Epigenetic Orchestration

**DOI:** 10.3390/microorganisms11122998

**Published:** 2023-12-16

**Authors:** Prabhakar Babele, Mukul K. Midha, Kanury V. S. Rao, Ajay Kumar

**Affiliations:** 1 Translational Health Science and Technology Institute, Faridabad 121001, India; prabhakar@thsti.res.in (P.B.); kanury.rao@gmail.com (K.V.S.R.); 2 Institute for Systems Biology, Seattle, WA 98109, USA; mkmidha@gmail.com

**Keywords:** temporal proteomics, *Mycobacterium tuberculosis*, LFQ proteomics, SWATH-MS, macrophage-like THP1 cells

## Abstract

Apart from being preventable and treatable, tuberculosis is the deadliest bacterial disease afflicting humankind owing to its ability to evade host defence responses, many of which are controlled by epigenetic mechanisms. Here, we report the temporal dynamics of the proteome of macrophage-like host cells after infecting them for 6, 18, 30, and 42 h with two laboratory strains (H37Ra and H37Rv) and two clinical strains (BND433 and JAL2287) of *Mycobacterium tuberculosis* (MTB). Using SWATH-MS, the proteins characterized at the onset of infection broadly represented oxidative stress and cell cytoskeleton processes. Intermediary and later stages of infection are accompanied by a reshaping of the combination of proteins implicated in histone stability, gene expression, and protein trafficking. This study provides strain-specific and time-specific variations in the proteome of the host, which might further the development of host-directed therapeutics and diagnostic tools against the pathogen. Also, our findings accentuate the importance of proteomic tools in delineating the complex recalibration of the host defence enabled as an effect of MTB infection. To the best of our knowledge, this is the first comprehensive proteomic account of the host response to avirulent and virulent strains of MTB at different time periods of the life span of macrophage-like cells. The mass spectrometry proteomics data have been deposited in the ProteomeXchange Consortium via the PRIDE repository with the dataset identifier PXD022352.

## 1. Introduction

Over the course of human history, *Mycobacterium tuberculosis* (MTB) has killed more people than any other bacteria. It is estimated that approximately one-fourth of the world’s population is infected by the pathogen, with only one new anti-tubercular drug approved by the Food and Drug Administration (FDA) for almost 40 years [1,2,3]. MTB is a respiratory pathogen that evolved thousands of years ago from environmental mycobacteria. Its prolonged co-existence with humans enabled it to be well-adapted and aware of the host defences. Aerosols from an infected individual are its primary means of transmission and only three bacilli are sufficient to establish infection [4]. Tissue-resident alveolar macrophages are infected by the bacilli within the lung interstitium and are the primary responders to MTB infection. Further immune response to infection through activation of adaptive immunity may take 2–8 weeks [5].

Delineating the host pathways that are perturbed in the presence of a virulent pathogen and are obligatory for disease progression will be critical for understanding the biology of infection. There is tremendous variability among different hosts, depending upon genetics, co-morbidities, as well as environmental factors. Only 5–10% of latent tuberculosis (TB) patients progress to active TB. Also, of all the Bacillus Calmette–Guérin (BCG)-immunized individuals, only around 50% are effectively protected from TB [6]. While the host is fully capable of countering the pathogenic bacilli, perturbations in host machinery play a significant role in the determination of clinical outcomes and pathogenicity. Several reports have demonstrated the involvement of epigenetic modulation of host gene expression, mostly via DNA methylation, alteration in the nucleosome, and binding of transcription factors to dampen the anti-bacterial responses [7]. Many intracellular pathogens alter the expression of enzymes involved in modifying histones in the host cell, such as histone deacetylase, methylase, and sirtuins. Such nucleosomal changes could be rescued by host-directed therapeutics that target these enzymes [8,9].

Additionally, different strains of MTB display a complex array of cellular functions under adverse conditions within the host and differently modulate host cellular processes to its advantage. While H37Ra and H37Rv are the common laboratory strains—where the former is an avirulent strain of MTB and the latter is a virulent strain, the other two MTB strains (BND433 and JAL2287) are the clinical virulent strains, such that BND433 is a single-drug-resistant strain and JAL2287 is a multi-drug-resistant (MDR) strain. Details regarding the pathogenesis, virulence, and persistence of clinical and/or drug-resistant strains are not well understood within the scientific community, leading to a lag in universally accepted vaccines against the disease [9]. Our study could be the Achilles heel for a better knowledge of TB pathogenesis and the simultaneous development of preventives and therapeutics. Recently, omics technologies have paved the way forward for rapid screening of the host as well as strain characterization, genome-wide comparison of different virulent strains of MTB, transcriptomics analyses of drug-sensitive and drug-resistant strains, and functional assays using proteomics [10,11,12]. 

Quantitative proteomics, in particular, is a powerful tool for deciphering the molecular details of host–pathogen interactions. All cellular processes are dictated by alterations in the protein machinery as the host modulates its defences via a subtle balance of its proteome compartment according to the virulence of the pathogen as well as the duration of infection. However, not much effort has been made to capture and evaluate the temporal dynamics of the host proteome in response to different strains of MTB infection. There is no comprehensive account detailing the landscape of host proteomic reprogramming over the duration of MTB infection. Our aim here was to snapshot the perturbations in the host proteome, manifested by infection with different strains of MTB bacilli, over four tandem infection-time windows (6, 18, 30, and 42 h). After the infection was set with respective MTB strains, we harvested the host proteome from the infected macrophage-like cells every 12 h and analysed it for strain-specific host proteome reprogramming. Earlier gel-based two-dimensional electrophoresis (DE) approaches identified differential proteomic profiles of strain-specific protein signatures [13,14]. With advancements in technology, large proteomic datasets can be generated using liquid chromatography (LC)–mass spectrometry (MS) in quick succession to elucidate complex biological systems [9]. However, proteomic reports on clinical strains or MDR MTB have been limited. In the present study, we used a macrophage-like THP1 cell line as the model for human macrophage after differentiating the cells with phorbol 12-myristate 13-acetate (PMA), followed by infection with either laboratory avirulent strain H37Ra, or virulent strains H37Rv, BND433, and JAL2287. Differentiated THP1 cells without any infection were the infection control set. Label-free data-independent acquisition (DIA)–sequential window acquisition of all theoretical mass spectra (SWATH)-based quantitative proteomics was used to map the host proteome at four tandem time points (6, 18, 30, and 42 h) in response to the infection. Our study could have theranostic potential by uncovering the complex mechanistic details and survival strategies of this obstinate pathogen highlighted through the reprogramming of a host’s cellular machinery.

## 2. Materials and Methods

### 2.1. Cell Culture

Different strains of MTB were cultured in Middlebrook 7H9 broth (Becton Dickinson Difco, Sparks, MD, USA) supplemented with 10% ADC (Becton Dickinson, Sparks, MD USA), 0.4% glycerol, and 0.05% Tween 80. Bacteria were harvested in the mid-log phase, resuspended in RPMI medium, dispersed by aspiration to break clumps, and quantified by the spectrophotometric method, as reported before [15]. For THP1 cell lines, RPMI 1640 (Gibco, Grand Island, NY, USA) supplemented with 10% FCS (Hyclone, Marlborough, MA, USA) and 1× penicillin (stock concentration = 100 U/mL) and streptomycin (stock concentration = 100 μg/mL) was used. Cells were grown and maintained at 37 °C with appropriate humidity and 5% CO_2_. A detailed methodology can be found in our earlier report [15]. 

### 2.2. Mycobacterial Infection and Proteomic Sample Preparation

PMA (5 ng/mL) was used for differentiating THP1 cells into macrophage-like cells by incubating for 48 h at 37 °C with appropriate humidity and 5% CO_2_. A total of 10 bacteria per macrophage-like cell were used for infection along with an uninfected control set. Overall, THP1 cells were differentiated into macrophage-like cells in the presence of PMA in 60 T-150 flasks. After differentiation, the flasks were divided into five batches of 12 flasks each. One batch was used as an uninfected control batch while the other four batches were infected with H37Ra, H37Rv, BND433, and JAL2287 MTB strains, respectively, at MOI10. At each of the decided harvest time points (6, 18, 30, and 42 h), infected cells from three flasks from each batch were trypsinized and collected by centrifugation at 1000 rpm and 4 °C. Cells were lysed on ice in the presence of a protease inhibitor cocktail (Pierce, Rockford, IL, USA) and benzonase (Merck, Darmstadt, Germany) in 8 M Urea, 200 mM Tris pH 8, 4% CHAPS, and 1 M NaCl. Total protein concentration was determined by the Bradford method, according to the manufacturer’s information. Protein samples were reduced, alkylated, and digested with trypsin, and prepared for LC-MS/MS as reported previously [16]. Before LC-MS, peptides were separated in the first dimension by SCX chromatography (5 μm, 300˚A, Sciex, Framingham, MA, USA). 

### 2.3. Data-Dependent Acquisition (DDA) and Data-Independent Acquisition (DIA) Using NanoLC-MS/MS

DDA sample files were used for spectral library generation, acquired using nanoLC-Ultra (Eksigent, Toronto, ON, Canada) online coupled to a TripleTOF 5600 mass spectrometer (SCIEX, Concord, CAN). Peptides were separated on a C18 high-resolution 200 cm (LCGC Sciences, Tokyo, Japan) column. The samples were spiked with 1x indexed retention time (iRT) reference peptides (Biognosys AG, Schlieren, Switzerland) and 200 femtomol β-galactosidase (as internal standard) prior to MS injection for RT calibration. The mobile phase for HPLC consisted of water/acetonitrile/formic acid (A, 98/2/0.2%; B, 2/98/0.2%) with a flow rate of 400 nL/min. A linear gradient from 5 to 50% of buffer B over the period of 275 min was used for peptide separation. The DDA parameters were: MS1 mass range 350–1250 *m*/*z* for 200 ms, 20 most intense precursors with charge states of 2–5 that exceed 100 counts/s were selected for MSMS using rolling collision energy, while MSMS spectra were collected from 200 to 1800 *m*/*z* for 70 ms. A 10 μm SilicaTip electrospray PicoTip emitter (New Objective) was used to inject peptides into the mass spectrometer. For DIA, using different isolation widths, a set of variable overlapping windows was constructed covering the mass range 350–1250 Da. The collision energy for each window was determined based on the appropriate collision energy set automatically with a spread of 5 eV. The total duty cycle was 4.02 s. 

### 2.4. Spectral Library Generation and Quality Control

The identification of proteins for library generation was performed via a database search in the UniProt Homo sapiens protein database (January 2015 release), with ProteinPilot 5.0.2 software with an in-built Paragon algorithm to obtain the spectral library. The search parameters were as follows: (1) sample type, identification; (2) cysteine alkylation, iodoacetamide; (3) digestion, trypsin; (4) instrument, TripleTOF 5600; (5) ID focus, biological modifications; and (6) search effort, thorough ID. A reverse database search strategy was adopted to estimate the global false discovery rate-fit (G-FDR-fit) for peptide identification. The in-house spectral assay library was evaluated using DIALib-QC [17]. Only those proteins meeting the 95% confidence, minimum of two unique peptides, and 1% FDR criteria were used for further analysis [18].

### 2.5. SWATH-MS and Data Processing

The spectral library generated by ProteinPilot was directly imported into the Spectronaut DIA software version X (Biognosys, Switzerland). The nonlinear iRT calibration strategy employed a dynamic window for both mass tolerance (MS1 and MS2) and to set up the extracted ion chromatogram (XIC) retention time (RT) window. Pre-processing of MS1 and MS2 calibration strategies was enabled. Decoy assays were dynamically generated. Identification employed the kernel density estimator, with precursor and protein identification results being subjected to a q-value filter of <0.01 and data filtering for q-value with local normalization. For quantification purposes, the averaging of MS2 ion peak areas from quantified peptides was performed to estimate protein peak areas. The iRT peptides were used in automatic calibration mode for spectral RT alignment between the library and SWATH datasets. Default parameter settings were applied along with these procedures. Additional parameter settings were used as the default.

### 2.6. Statistical and Bioinformatics Analyses

All the experiments were carried out independently in triplicate and the results are expressed as mean ± standard error of the mean (SEM). GraphPad Prism 8 was used for multiple group comparisons using one-way analysis of variance (ANOVA) and the Bonferroni test. Statistical analyses were performed using a *t*-test (Sigma Stat, San Jose, CA, USA) to compare the relative expression of proteins in different groups. Functional classification of all differentially expressed proteins (DEPs) was carried out using the online Gene Functional Classification Tool, DAVID Bioinformatics Resources 6.8, NIAID/NIH, accessed on 11 January 2023 (https://david.ncifcrf.gov) [19]. Interacting partners for DEPs were predicted using the Search Tool for the Retrieval of Interacting Genes/Proteins (STRING) 9.0 database [20].

## 3. Results

### 3.1. Differential Proteome Analysis of Uninfected and Infected Macrophage-like Cells

Our study describes the changes in protein intensities in the host proteome in response to infection by different strains of MTB at four time points in the macrophage-like THP1 cell line model. To estimate the abundance of the identified proteins, Spectronaut software (version X) was used to process the DIA/SWATH data. This method takes into consideration reconstructing XICs from the fragment ion intensities of the MSMS spectra [21]. Comparative proteomics was performed for all datasets with three biological replicates; three injection replicates were prepared for analysis of peptide mixture obtained from each set (total of nine for one dataset). Among the 1802 unique macrophage proteins identified by DDA in the composite proteome, we were able to quantify 1304 proteins after the application of label-free quantitation (LFQ) (Table S1). To cross-compare the reproducibility of results across the biological replicates, a correlation matrix was generated, and we found a narrow variability in standard variation and normal distribution, indicating a good consistency between the measurements. In a given dataset, proteins showing altered abundance (average ratio test/control either above 1.50 or below 0.66 compared with the mean (1.00)) and a *p*-value < 0.05 were considered as DEPs and used for further analysis. The MSMS intensities of proteins spanned a dynamic range of four orders of magnitude with the majority of all identified proteins present in the low abundance range (Figure S1). 

### 3.2. Temporal Proteomic Response of Macrophages against MTB

The host proteome is significantly reprogrammed after infection with a pathogen and such perturbations manifest further with increasing duration of infection. Therefore, it was pertinent to assess the global proteomic landscape of MTB-infected macrophages as the pathogen manifests itself within the host. Host cells were harvested and lysed at four tandem time points (6, 18, 30, and 42 h) after infecting them with different strains of MTB and processed for label-free quantitative proteomics analysis. A bird’s eye view of the time-dependent dynamics of the macrophage proteome in response to different MTB strains (Table 1) highlights the adaptability and pathogenicity of the world’s most successful pathogen [22]. 

With the aim of revealing the putative host markers against MTB virulent strains (whether of lab or clinical origin), we divided the DEPs of infection at each of the aforementioned time points into two groups—the first group encompassed all the shared proteins showing dysregulation after infection by any of the three virulent strains (i.e., H37Rv-BND433, H37Rv-JAL2287, BND433-JAL2287, and H37Rv-BND433-JAL2287), whereas the second group consisted of unshared DEPs found uniquely in a particular strain (H37Ra, H37Rv, BND433, and JAL2287) without overlapping with other datasets. The common virulent DEPs were important because they were altered as a consequence of infection with any of the virulent MTB strains, but not by the avirulent strain, and reflected a convergent host defensive machinery against MTB pathogenesis. Moreover, the unique unshared DEPs contributed additional biological functions elaborated in response to specific MTB pathogenic infections, thereby reflecting a range of responses in macrophages against H37Rv, BND433, and JAL2287. The selected time windows for investigating the perturbations in host response to MTB ensured a comprehensive collection of the host proteome every 12 h of infection up to 42 h after the initial MTB infection was set for 6 h. As seen here as well as in previous studies, significant cell death occurred in cells infected with virulent strains after 42 h of infection and therefore no further time points were collected.

Early-phase response is characterized by transient oxidative stress, intermediate filament re-organization, and protein import to the nucleus: A total of 46 DEPs were found common to all three virulent strains at the early stage (6 h) of infection of macrophages; of these, 31 proteins were up-regulated while 14 proteins were down-regulated (Figure S2). To seek the integrative functions of infection-induced proteins at 6 h in macrophage, all the common virulent DEPs were classified by gene ontology terms separately for up- and down-regulated proteins. The differentially modulated proteins were over-represented from a background reference set of human proteins with a positive fold enrichment (*p* < 0.05) and were assigned different gene ontology (GO) terms by the gene ontology consortium [23]. The top-seeded terms with respect to the gene ontology (GO) biological process (BP) were intermediate filament (IF) organization (17%), epithelial cell differentiation (17%), peptide cross-linking (10%), response to oxidative stress (10%), and protein localization and oligomerization (7%) (Figure S3). To obtain further functional enrichment and pathways for macrophage proteins, these DEPs were also sorted into cellular components, molecular functions, and Kyoto Encyclopedia of Genes and Genomes (KEGG) pathways (*p* < 0.05) as given in Figure S4. Notably, the top gene ontology (GO) molecular function (MF) terms were protein (93%) and RNA binding (43%) and ubiquitin protein ligase activity (28%), and the top gene ontology (GO) cellular component (CC) terms were intermediate filament (13%) and mitotic spindle (14%), suggesting a reshuffling of macrophage internal skeleton by scaffold proteins early in the MTB infection [24].

Thioredoxin, thioredoxin-dependent peroxide reductase, BolA-like protein 2, glutamate–cysteine ligase, and cytosolic NADP-isocitrate dehydrogenase are all integral to the “oxidative stress” pathway which was found in the top 10 enriched pathways of the initial phase of infection (Figure S3). The thioredoxin-dependent peroxide reductase (H37Rv_6_ 1.6×, BND433_6_ 1.6×, JAL2287_6_ 2.8×) and cytosolic NADP-isocitrate dehydrogenase were up-regulated (H37Rv_6_ 2.6×, BND433_6_ 2.8×, JAL2287_6_ 1.7×) initially (Table S2) with thioredoxin (BND433_18_ 0.6×, JAL2287_18_ 0.6×), bolA-like protein 2 (H37Rv_18_ 0.2×, BND433_18_ 0.4×), and glutamate–cysteine ligase (BND433_18_ 0.4×, JAL2287_18_ 0.3×) down-regulated at the second time point (Table S3)—indicating an initial host defence followed by the subjugation of the host oxidative machinery. Increased oxidative stress and abundance of reactive oxygen species (ROS) and reactive nitrogen species (RNS) have previously been linked to the pathogenesis of the *M. tuberculosis* H37Rv strain [25]. Importantly, stress granule assembly was also observed to be an enriched function at 18 h of infection with the virulent strains. Stress granules are membrane-less compartments that store mRNAs and proteins, such as stalled translation pre-initiation complexes, in response to stress. Cold shock domain-containing protein E1 (H37Rv_18_ 0.5×, BND433_18_ 0.6×, JAL2287_18_ 0.3×) and cold-inducible RNA-binding protein (BND433_18_ 0.5×, JAL2287_18_ 0.6×) were both decreased while Ras GTPase-activating protein-binding protein 2 was shown to be down-regulated in H37Rv (H37Rv_6_ 0.6×, H37Rv_18_ 0.3×), BND433 (BND433_18_ 0.5×), and JAL2287 (JAL2287_6_ 0.4×) infection but not with JAL2287 (JAL2287_18_ 2.2×) [26] (Tables S2 and S3). TAR DNA-binding protein 43 (TDP-43) is another important macrophage protein thought to associate with stalled ribosomes localized to stress granules and contributes to host cell survival in response to pathogen-driven oxidative insult [27]. All these proteins help prevent the successful establishment of MTB within macrophages; however, their down-regulation explains how the infection prevails and the host succumbs to the mycobacterial pathogenesis. 

From the GO term enrichment analysis, “intermediate filament organization” was, at the first time point (Figure S3), one of the most highly enriched biological processes with proteins like procollagen-lysine 2-oxoglutarate 5-dioxygenase 1 (H37Rv_6_ 1.7×, BND433_6_ 1.6×), epithelial keratin-2e (BND433_6_ 2.6×, JAL2287_6_ 4.6×), etc. (Table S2), all up-regulated at this time point and related terms featuring in all four enrichment categories. The IF proteins were not found to be an enriched term at the subsequent time points. However, exceptions to this were other cytoskeletal processes, i.e., actin filament and microtubule organization, with proteins thymosin, coronin, tubulin, dynactin, tropomodulin, cofilin, profilin, etc., showing a variety of expression levels in unique unshared DEPs of each dataset (Table S1). Thus, it would seem that a transient IF organization was induced initially—possibly in response to oxidative stresses—and then mostly, but not entirely, silenced. Moreover, the unique unshared DEPs found in macrophages infected with H37Rv, an explicit down-regulation of a number of proteins critical to cell adhesion, such as integrin alpha-M (H37Rv_6_ 0.6×), integrin alpha-X (H37Rv_6_ 0.5×), and collagen alpha-1(VI) chain (H37Rv_6_ 0.6×), were also observed (Table S2). This reorganization of internal cellular structures is postulated to hinder the effective clearance of pathogens [28]. 

“Protein import to nucleus” was seen as an enriched biological process at the early time point of 6 h (Figure S3) with importin-5, involved in protein trafficking to the nucleus, up-regulated (BND433_6_ 2.0×, JAL2287_6_ 2.0×). However, in the later infection stage, we saw a marked decrease in its expression level (H37Rv_42_ 0.5×, BND433_42_ 0.6×, JAL2287_42_ 0.6×) (Table S2) together with importin subunit beta-1 (H37Ra_42_ 0.6×, H37Rv_42_ 0.5×, BND433_42_ 0.6×, JAL2287_42_ 0.6×). Thus, there is clear evidence for an immediate dysregulation of proteins involved in protein trafficking to the nucleus, the site of epigenetic moulding of the telomere, nucleosome, and splicing, with MTB infection. In addition to TB, importin-5 has also been shown to play an important role in diseases such as cancer and viral replication [29]. 

Further, 22, 41, and 97 proteins were also uniquely dysregulated (*p* < 0.05) by infection with H37Rv, BND433, and JAL2287, respectively, after 6 h (Figure S2); among the DEPs uniquely found in macrophages against H37Rv, 25% of proteins belonged to chromatin organization, 35% to innate immune response, 18% to cell adhesion, and the remainder to other miscellaneous functions (Figure S3). Likewise, functional analysis of host DEPs infected with the BND433 clinical strain classified 10 proteins under pre-replicative and nucleosome assembly (25%) and 4 proteins under translation (10%). These annotations further revealed that during JAL2287 infection, the functional categories that were largely composed of up-regulated proteins included ATP biosynthetic processes (9%) and TNF-mediated signalling (5%), such as cell differentiation, apoptosis, and inflammation (11/78), while 19% of proteins which were down-regulated came from mRNA splicing (4/21) (Figure S3). Overall, the results present a distinct set of host proteins modulated under MTB infection stimulating the intracellular environments.

**Figure 1:**
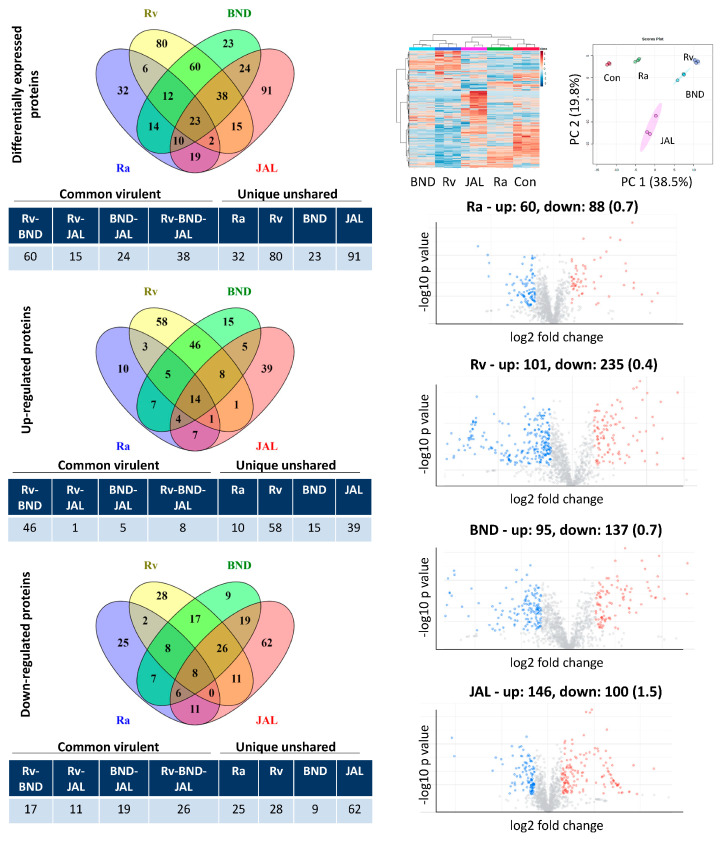
Label-free DIA-SWATH analysis of macrophage-like cell proteome after infection with different strains of *M. tuberculosis* at 18 h of infection. Host cells were harvested and subjected to LC-MS/MS analysis following protein extraction and trypsin digestion. Left panel: The number of differentially expressed proteins detected in each dataset. The differentially expressed proteins were grouped into common virulent proteins, if they were shared between any of the three virulent strains (H37Rv, BND433, and JAL2287), and unique unshared proteins, if they were not shared among them. Right panel: Unsupervised hierarchical clustering and principal component analysis of the protein profiling of THP1 cells. Volcano plots with up- and down-regulated proteins (up/down ratio in parentheses) for each dataset are also shown. Con: control; Ra: H37Ra; Rv: H37Rv; BND: BND433; JAL: JAL2287.

**Figure 2:**
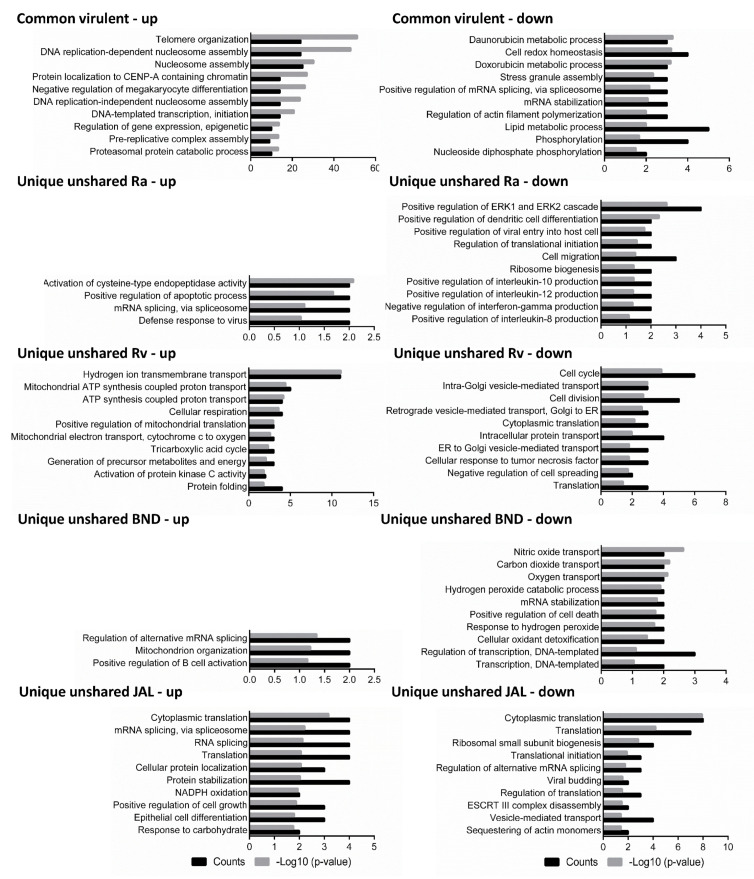
Biological processes were predicted for the differentially expressed proteins of macrophage-like cells after infection with different strains of *M. tuberculosis* at 18 h of infection. The differentially expressed proteins were grouped into common virulent proteins, if they were shared between any of the three virulent strain infections (H37Rv, BND433, and JAL2287), and unique unshared proteins, if they were not shared among them. Left panel: up-regulated proteins; right panel: down-regulated proteins. Ra: H37Ra; Rv: H37Rv; BND: BND433; JAL: JAL2287.

**Figure 3:**
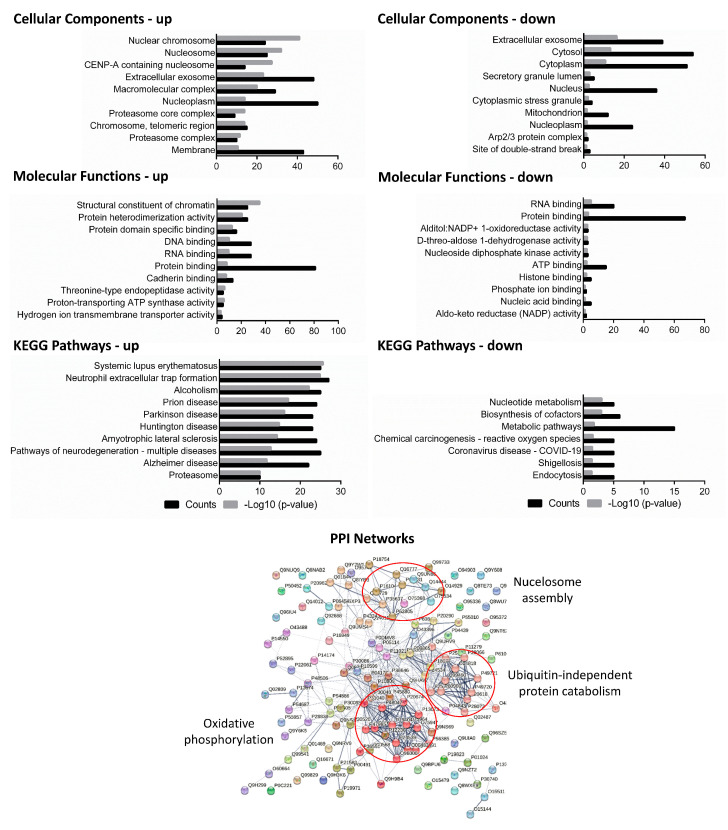
Cellular components, molecular functions, and KEGG pathways were predicted for the common differentially expressed proteins of the host that were shared between any of the three virulent strains (H37Rv, BND433, and JAL2287) after infection at 18 h of incubation. Upper left panel: up-regulated proteins; upper right panel: down-regulated proteins. Lower panel: protein–protein interaction (PPI) networks of protein interactions with Markov clustering in circles were also constructed. Nodes in the diagram represent proteins, and edges represent protein–protein interactions between the nodes. PPI: protein–protein interaction.

Intermediary and late THP1 response reveals concordant epigenetic modulation, ATP metabolism, intracellular protein transport, and vesicular trafficking: Principal component 1 accounting for the majority of variations (approximately 45%) in intermediary and late responses appeared to cluster samples primarily in the infection group (Figure 1), with more advanced time points having a larger separation (Figures S5 and S6). From the GO term enrichment analysis, 18, 30, and 42 h had similar biological processes (Figure 2 and Figure 3; Figures S7–S10), reflecting a somewhat consensus host response at intermediary- and late-stage infections. Of the total 137, 79, and 103 macrophage common proteins showing differential expression at 18, 30, and 42 h, respectively, after infection with virulent MTB strains (Figure 1; Figures S5 and S6), the up-regulated common virulent proteins identified in the proteome as part of this study were distributed over all the functional categories. The majority of the proteins belonged to telomere organization (27%), nucleosome assembly (29%), epigenetic regulation of gene expression (11%), and protein localization to CENP-A (16%), constituting 60% of the total identified proteome (Figure 2; Figures S7 and S9). Further, proteins involved in ATP metabolism (7%) and transcription initiation (16%) were also identified as important contributors to the up-regulated biological processes (Figures S7 and S9). Similarly, 73, 15, and 40 down-regulated common virulent proteins (Figure 1; Figures S5 and S6) were detected at the listed three time points, respectively, with the highest numbers related to actin filament assembly (5%) and protein localization and targeting (9%) (Figures S7 and S9). 

Among the top 10 enriched biological processes, “telomere organization and nucleosome assembly” features at all three later time points with histone H1.10 (BND433_42_ 1.8×, JAL2287_42_ 1.8×), histone H2AX (H37Rv_18_ 1.6×, BND433_18_ 1.5×, H37Ra_42_ 1.8×, H37Rv_42_ 2.1×, BND433_42_ 2.1×, JAL2287_42_ 2.7×), histone H3.1 (H37Rv_18_ 2.3×, BND433_18_ 2.2×, H37Rv_30_ 1.8×, BND433_30_ 1.8×, H37Rv_42_ 2.4×, BND433_42_ 2.4×, JAL2287_42_ 1.6×), histone H4 (H37Rv_18_ 2.1×, BND433_18_ 1.6×, JAL2287_30_ 2.2×, H37Rv_42_ 2.5×, BND433_42_ 1.7×, JAL2287_42_ 1.8×), heterochromatin protein 1-binding protein 3 (H37Rv_18_ 1.5×, BND433_42_ 1.7×, JAL2287_42_ 1.5×), acidic leucine-rich nuclear phosphoprotein 32 (H37Rv_18_ 0.6×, BND433_18_ 0.6×, H37Rv_30_ 0.4, BND433_30_ 0.5×, JAL2287_30_ 1.8×, H37Ra_42_ 0.4×, H37Rv_42_ 0.5×, BND433_42_ 0.5×, JAL2287_42_ 0.6×), and prohibitin 1 (H37Rv_18_ 2.1×, H37Rv_30_ 1.8×, BND433_30_ 1.5×) all dysregulated as early as the second time point (Tables S3 and S5). It would thus appear that MTB engaged in host epigenetic orchestration to evade host immune defences, including antigen presentation, apoptosis, autophagy, cytokine production, immune cell mobility, and phagocytosis. Acidic leucine-rich nuclear phosphoprotein 32 family member B is an interesting protein, exhibiting histone chaperone properties, able to recruit histones to certain promoters, and thus regulating the transcription of specific genes. This protein was consistently missing in H37Ra datasets (absent at 6, 18, and 30 h), consistently present in the virulent strains at all investigated time points (Figure S11, Table S6), and showed steadily decreasing expression in the virulent strain-infected macrophage samples over time. It would thus appear to be the most straightforward one of the several explanations that extensive epigenetic modulation of the host genome lies in the gradual depletion of this transcriptional regulator protein. Furthermore, actin-related protein 2/3 complex (ARP2/3) (H37Rv_18_ 0.6×, BND433_18_ 0.6×, JAL2287_18_ 0.5×, H37Rv_30_ 0.6×, JAL2287_30_ 2.0×, H37Rv_42_ 0.6×, BND433_42_ 0.6×, JAL2287_42_ 0.6x) (Tables S3 and S5) was also found consistently at all three later time points. In addition to its role in the cytoplasmic cytoskeleton, ARP2/3 also promotes homologous recombination repair in response to DNA damage by promoting nuclear actin polymerization, thereby regulating gene transcription and repair of damaged DNA [30].

Following infection with virulent strains, the “proton transport and ATP metabolism” was observed to be up-regulated relative to controls at each of the time points: with 22 proteins including cytochrome c oxidase 2 (H37Rv_18_ 1.8×, H37Rv_30_ 1.9×, H37Rv_42_ 1.8×, BND433_42_ 1.8×), stomatin-like protein 2 (H37Rv_30_ 1.9×, BND433_30_ 1.9×), cytochrome b-c1 complex Rieske (H37Rv_18_ 1.7×, BND433_18_ 1.9×, H37Rv_42_ 2.4×, BND433_42_ 1.8×), V-type proton ATPase E1 (H37Rv_42_ 1.6×, BND433_42_ 1.6×), etc., known to be part of the process identified as up-regulated at one time point at least, and all following similar expression profiles across different time points (Tables S3 and S5). Via GO-BP functional enrichment analysis we could also find that after infection with all three virulent strains, the set of maximally modulated macrophage proteins predominantly belonged to ATP synthase activity (20% for H37Rv_18_, 7% for JAL2287_30_, and 6% for BND433_42_) (Figure 2; Figures S7 and S9). Our findings deserve further discussion owing to the differential expression patterns of these proteins and altered energy metabolism at the subsequent time points.

At the third time point, there was greater dysregulation of “protein transport and vesicular trading” with four proteins significantly down-regulated: signal recognition particle 9 kDa protein (H37Rv_30_ 0.2×, BND433_30_ 0.4×), DDRGK domain-containing protein 1 (H37Rv_30_ 2.0×, BND433_30_ 2.3×, JAL2287_30_ 0.5×), and vesicle-associated membrane protein-associated protein A (H37Rv_30_ 0.5×, BND433_30_ 0.6×) (Table S4). SRP9 and SRP14 are both typically involved in the retardation of ribosome elongation of signal-peptide-containing proteins before their engagement with the translocation machinery in the endoplasmic reticulum [31]. Additionally, multiple DEPs were found to be involved in intracellular protein transport in a unique unshared category when macrophages were infected with either H37Rv or JAL2287 for 18 h, such as alpha-soluble NSF attachment protein (H37Rv_18_ 0.6×), coatomer subunit alpha (H37Rv_18_ 0.6×), coatomer subunit beta’ (H37Rv_18_ 0.6×), charged multivesicular body protein 5 (JAL2287_18_ 0.5×), coatomer subunit delta (H37Rv_18_ 0.6×), Ras-related protein Rab-2A (JAL2287_18_ 0.6×), vesicle-associated membrane protein 2 (JAL2287_18_ 0.6×), vacuolar protein-sorting-associated protein VTA1 homolog (JAL2287_18_ 0.5×), clathrin interactor 1 (JAL2287_18_ 0.6×), and alpha-centractin (JAL2287_18_ 0.6×) (Table S3). A thorough down-regulation of these structural proteins explains the shutting down of the host protein machinery altogether. At 30 h post-infection with H37Ra bacilli, the expression levels of 131 macrophage proteins significantly increased and 14 proteins decreased (Figure S5), which is associated with avirulent strain infection. Here, some proteins related to protein targeting to mitochondria, but not endoplasmic reticulum (ER), were also seen to be up-regulated—ubiquitin-conjugating enzyme E2 L3 (H37Ra_30_ 1.5×), ubiquitin-conjugating enzyme E2 D3 (H37Ra_30_ 1.5×), Ras-related C3 botulinum toxin substrate 2 (H37Ra_30_ 2.8×), and puromycin-sensitive aminopeptidase (H37Ra_30_ 1.6×) (Table S4). A similar trend was seen at the early infection stage (6 h), where the expression levels of 34 macrophage proteins significantly increased and 70 proteins decreased in the H37Ra dataset (Figure S2). Two proteins related to protein targeting to the membrane were found with a 4.6-fold change difference for translocon-associated protein subunit alpha and a 1.7-fold change for signal recognition particle 14 kDa protein (Table S2). This implies an extensive role of protein targeting to various cellular structures not only in virulent MTB strains but also in the avirulent strain.

Proteins involved in the regulation of “protein stability” were also prominent: TDP-43 (H37Rv_30_ 0.5×, BND433_30_ 0.6×, JAL2287_30_ 3.7×), lysosome-associated membrane glycoprotein 2 (BND433_30_ 0.1×, JAL2287_30_ 2.9×), apoptosis-associated speck-like protein (ASC) (BND433_30_ 0.6×, JAL2287_30_ 2.0×), DDRGK domain-containing protein 1 (H37Rv_30_ 2.0×, BND433_30_ 2.3×, JAL2287_30_ 0.5×), vacuolar protein-sorting-associated protein 35 (JAL2287_6_ 1.6×, H37Rv_42_ 1.6×), CD81 antigen (BND433_18_ 1.7×, H37Rv_42_ 1.9×), and serotransferrin (BND433_6_ 1.9×, JAL2287_6_ 1.7×, H37Rv_18_ 1.8×, H37Rv_30_ 1.7×, BND433_30_ 1.9×, BND433_42_ 1.8×) were all dysregulated (Tables S2 and S5). This is notable as the TDP-43 is shown to regulate the splicing of many non-coding and protein-coding RNAs including proteins involved in neuronal survival [32]. The dysregulation of this protein and its related accessories may offer further evidence of protein and mRNA stability in intermediary and late responses to bacterial infection.

When considering the proteome from the avirulent strain dataset, we found a generalized classic immune response elaborated by macrophages under H37Ra infection. The expression levels of proteins involved in dendritic cell differentiation, IL-8, IL-10, IL-12, and IFN-gamma production in macrophages, were noted exclusively (6–18 h) (Figure 2 and Figure S3). There were 34 and 10 macrophage proteins with increased expressions and 70 and 25 proteins with decreased expressions after H37Ra infection at 6 and 18 h, respectively (Figure 1 and Figure S2). Such immune response is altogether absent in the cases of virulent strain (H37Rv, BND433, and JAL2287) infections from 18 h onwards (Figure 2, Figures S7 and S9), perspicuously stipulating the shutting down of host immune genes most likely by epigenetic remoulding (Table 1).

To further elucidate the interaction map of common host DEPs against virulent bacilli, protein interaction networks were constructed with STRING 12.0 and sub-networks were created with MCL clustering. There were 20% macrophage proteins related to oxidative phosphorylation, 17% proteins correlated with ubiquitin-independent protein catabolism, 9% proteins with post-translational modifications (PTMs), and there were 7% host proteins related to nucleosome assembly, indicating that these host DEPs and their interaction clusters at intermediary and late infection stages may be involved in the virulence of MTB infection (Figure 3, Figures S8, and S10). Notably, among the nucleosome assembly clusters, the most obvious proteins were regulators of chromosome condensation and other histone proteins, corroborating the earlier results of gene ontology. Oxidative phosphorylation has been a consistent protein cluster found at all time points with all MTB strains, signifying a core response in the form of energy balance and metabolic shuffling. 

Taken together, these functional annotations revealed that infection with MTB virulent strains could lead to changes in the expression of macrophage proteins mostly associated with genetic make-up, gene expression, energy metabolism, and protein stability, which was different from H37Ra infection at different time points. However, the 42 h time point could also represent the time at which proteomic changes that accompany ageing were also expected to be observable. This study confirmed that host macrophages infected with avirulent or virulent MTB strains exhibit divergent biological processes while significant cross-talk also existed between them.

## 4. Discussion

A great deal of our current knowledge regarding mycobacterial pathogenesis in a host is based on the proteomic elucidation of the host as well as pathogen using various proteomic tools, such as LFQ, stable isotope labelling by amino acids in cell culture (SILAC), tandem mass tag (TMT), etc. [33,34,35]. However, both pathogen and host change their biochemical and physiological states, driven by their proteomic machinery, as the infection cycle stretches out, highlighting the importance of temporal proteome dynamics study. Within this context, our data shed new light on different molecular mechanisms behind the cross-talk of MTB and host contributing to the pathogenesis of mycobacteria in a macrophage-like cell line. We used label-free SWATH-MS to profile the proteins in THP1 cells infected with H37Ra, H37Rv, BND433, and JAL2287 strains and extracted the host proteins at four tandem time points (6, 18, 30, and 42 h) after infection, with untreated cells as an infection control. Overall, we found a striking increase in the downregulation of most of the host proteins upon MTB infection as the infection time dragged out. 

Inhibition of phagolysosomal fusion, oxidative stress, autophagy machinery, antigen presentation, and alteration in T-cell immunity are a few of the host mechanisms that are successfully evaded by MTB. And the most remarkable fact about MTB as an intracellular pathogen is its ability to alter the host epigenome in order to subvert some of the host’s defence mechanisms [8]. Epigenetics is the alteration in gene function without any change in the nucleotide sequences of the genes, and mostly involves either DNA methylation or histone post-translational modifications. MTB-induced epigenetic changes could affect the host cell by activation or suppression of key immune genes to promote its survival and topple the antibacterial strategies of the host. Among the DEPs of macrophages found in our analysis between infections with virulent and avirulent MTB strains, almost two-thirds of the proteins were related to telomere organization, nucleosome assembly, gene expression, and protein localization to CENP-A. Several epigenetically relevant proteins, such as histone-modifying enzymes, nucleosome remodelling complexes, and histone chaperones required for eukaryotic transcription are identified as DEPs in this study. 

Chromatin dynamic architecture is vital for the proper functioning of cellular processes where numerous nuclear proteins, including histones, have critical roles to play. Heterochromatin protein 1 binding protein 3 (HP1BP3) is related to the linker histone H1 family conferring a higher-order organization to chromatin. Thus, any change in its level would result in altered gene expression. The HP1 family of heterochromatin proteins has been shown to induce mitotically heritable epigenetic gene silencing of euchromatic genes when placed adjacent to a block of silent heterochromatin in a mammalian cell culture model [36]. Both HP1 and HP1BP3 are examples of proteins that bridge the gap between epigenetic modifications and chromatin structure, ultimately influencing gene expression patterns and cellular functions. HP1BP is a crucial mediator assisting the binding of HP1 to the methylated H3 tail for the subsequent establishment of a repressive chromatin environment for gene expression. Consistent upregulation of HP1BP3 in our infection groups suggests an aggrandized phenomenon of epigenetic gene suppression of the targeted immune-responsive genes. A study found 60% mortality in the pups of Hp1bp3−/− mice within 24 h of birth, with the surviving animals exhibiting a lifelong 20% growth retardation, propounding a precise regulation in its expression level necessary for survival and growth [37].

Acidic nuclear phosphoprotein 32B (ANP32B) has been found to bind transcription factors and is a well-known chaperone assisting the incorporation/eviction of histones to the promoter region. An early report found that ANP32B is recruited to specific regions of DNA in order to suppress gene expression in a promoter-specific manner [38]. One specific area where ANP32 might be involved in epigenetic modification is the modulation of histone acetylation. ANP32A has been suggested to bind directly to histone tails, preferentially to unmodified histone H3 tails, and inhibits histone acetyltransferase activity in acute myeloid leukaemia, as suggested by Yang et al. [39]. Although they did not perform differential proteomic analysis, they demonstrated that increased chromatin accessibility was associated with changes in gene expression. Our data are generally consistent in terms of increased activity of ANP32B with subsequent epigenetic amendments in the host genome following MTB infection. Certainly, ANP32 proteins provide plenty of opportunity to rationalize the epigenetic evidence, not only in cancer but also in infectious diseases.

Prohibitins (PHB1 and PHB2) are pleiotropic proteins with variegated functions depending on their cellular compartment and interactome. While the exact role of PHB1 in epigenetics is not as extensively studied, it is known to interact with various proteins and complexes that play a role in chromatin structure and gene expression regulation. In the cytosol, PHB1 interacts with histone deacetylase 4 (HDAC4) and prevents its escort to the nucleus; high expression of PHB1 leads to a low level of nuclear HDAC1 and its associated epigenetic changes [40]. A significant differential change in PHB1 concentration after mycobacterial infection in the host substantiates epigenetic deregulation caused by histone deacetylases. Additionally, PHB1 flocks together with PHB2 at the inner mitochondrial membrane to smoothen the mitochondrial functions, such as mitophagy, oxidative phosphorylation, apoptosis, etc. The differential countenance of PHB1 together with cytochrome c oxidase, stomatin-like protein, and cytochrome b-c1 complex pointed to a link between epigenetic distortion and mitochondria functionalities in the great white plague disease or tuberculosis. Recently, Matsumura et al. explained how the host PHB2 undergoes reduction by mycobacterial protein PE_PGRS30 in murine alveolar macrophage mitochondria leading to its dysregulation and apoptosis [41]. 

By interacting with histone modification enzymes and impacting the positioning of nucleosomes along the DNA, histones have been suggested to play a role in epigenetic regulation. In our study, many histone proteins were identified showing dysregulation upon virulent MTB infection. This is consistent with previous reports that also demonstrated an increase in host epigenetic bustle upon MTB infection. Mycobacterial enhanced intracellular survival (Eis) protein Rv2416c has been shown to add acetyl groups to the free histones, inhibiting the induction of several immune function genes, including class II trans-activator (CIITA), which regulates the class II MHC and ERK1/2-JAK pathways, thereby leading to compromised function of immune cells [8,42]. Another histone, H2AX, well known for its epigenetic modification by phosphorylation, acetylation, and ubiquitination in DNA damage, has demonstrated similar epigenetic events contributing to the development of active pulmonary TB [43,44]. Jose et al. have isolated Rv3423.1 protein from the chromatin of human macrophages infected with H37Rv, which is a novel histone acetyltransferase that acetylates histone H3 at the K9/K14 positions [45]. Chen et al. reported histone H3K14 hypoacetylation and H3K27 hypermethylation along with HDAC1 up-regulation and KDM6B down-regulation, which are associated with active pulmonary TB disease in blood leukocytes [46]. Madden et al. used transcriptomic profiling by RNA-seq to demonstrate changes in the expression of genes involved in type I interferon signalling pathways during MTB infection [47]. Our data are generally consistent with studies by Madden et al., where MTB, after 18 h of infection, triggered a marked reduction in immune-related genes, unlike what was seen at 6 h. Indeed, the reshaping of chromatin structure and gene repression is the target of many mycobacterial secretory proteins which in turn have moonlighting functions on the host immune genes. Therefore, in the present study, we captured a complementary representation of global changes in chromatin layout by identifying chromatin modellers through proteomic tools. Understanding how histones contributed to nucleosome and gene expression could provide further insights into their roles in epigenetic processes and cellular functions. 

Pathways such as ‘cell motility’, ‘protein transport’, and ‘protein stability’ were also evocative in the infected groups, indicating that the disequilibrium of proteins in these pathways, that we and others have observed, occurred following MTB infection [35]. We uncovered a small subset of proteins related to motility and transport that were both the most significant and highly changed DEPs, suggesting a discomposure in phagocytic processes upon MTB infection. While the primary role of the ARP2/3 complex is in the motile activities of the cell that require dynamic actin filament assembly, like cytokinesis, cell migration, endocytosis, vesicle trafficking, etc., its potential involvement in mycobacterial pathogenesis has also been reported [48]. Ghosh et al. reported that mycobacterium Rv1626 interacts with the ARPC4 subunit of ARP2/3 and a subsequent 6-fold down-regulation of Rv1626 gene expression was observed in ARPC4-expressing H37Rv bacilli [49]. A similar trend was also seen in our study where a concordant down-regulation of ARP2/3 was observed after 18 h of H37Rv infection indicating a scarred host cytoskeleton in its milieu making the macrophage environment habitable and impeding phagosome maturation. A complementary study found that a functional block of Ras-related proteins regulate the mycobacterial phagosome maturation stages in corroboration to our data where the Rab-2A level was seen to subside [50]. A thorough up-regulation of serotransferrin immediately after MTB infection and the presence of transferrin receptors on endosome-derived phagosomes in MTB-infected macrophages suggested an increased and dynamic nature of vesicular trafficking inside the host, as already reported earlier [51]. Esterhuyse et al. used blood transcriptional module enrichment analysis of differentially expressed genes and revealed disease-specific changes in cytoskeletal remodelling, distinguishing latent MTB infection and active TB sample sets [35]. Many of the subsets annotated in our study were consistent with identified terms in other studies, such as coronin, which is an important protein for the survival of MTB and is actively accumulated by living mycobacteria on phagosomal membranes [52]. Our study shows that phagosome maturation arrest in MTB-infected macrophages occurs due to genome-wide changes in chromatin accessibility together with the subversion of vesicular transport and protein stability, and that coatomer, Rab-2A, VAMP-2, centractin, TDP-43, lysosome-associated membrane glycoprotein 2, etc., could influence a subset of these signatures. 

Differential expression of several proteins in the subset that we identified is known to promote the MTB burden, highlighting the relevance of the current study in pathogenesis. Significantly decreased expression of ASC, SRP9, and SRP14 facilitates MTB growth. ASC specifically has been observed to be essential for IL-1β production and the development of effective host immunity [53]. Its abnormal concentration suggests a deviant host immune response. Expression of SRP9 and SRP14 is also significantly associated with unfolded protein response, ER stress, and MTB burden in macrophages [54]. 

Mycobacteria have been elusive for centuries, and therefore, the development of novel host-directed therapeutics is of utmost importance for the treatment of TB. Host-directed therapeutic development could be a promising area in this regard which targets host gene expression processes dodging the frame-up of the host cell machinery by MTB. Numerous studies have provided evidence of the epigenetic effect of MTB infection in macrophages, which can be considered as an epigenetic signature of TB disease. However, the temporal dynamics of host response to MTB infection remain a secret agenda. In this regard, we demonstrated that significant changes to chromatin structure are induced by a multitude of proteins following MTB infection with a lag, in which the pathogen rejigs its milieu for the ensuing protein incursion to the nucleus and epigenetic assault. Several identified proteins in this study have never previously been associated with MTB pathogenesis and could provide a novel way to develop biomarkers or targeted adjunct therapy against these identified proteins and pathways. This temporal proteomics study represents the most extensive insight into macrophage response against four different MTB strains reported to date, allowing the simultaneous identification of thousands of proteins undergoing dysregulation.

## 5. Conclusions

Findings from our study clearly highlight the manipulation of the host proteome by a mycobacterium to facilitate its personal survival and manifestation. Virulent MTB appears to consistently engage in host epigenetic orchestration, over the entire duration of infection, to evade host immune defences through ANP32B throughout the extended infection duration and ARP2/3 proteins during the latter half of the infection cycle. Our findings could have implications for host-directed TB diagnostics and therapeutics. The MTB strain-specific differential response of host proteins could provide deeper insights into the complex dynamics of host–pathogen interactions. The expansive variations found between the protein profiles of macrophages infected with avirulent and virulent strains and at different time periods suggest the existence of a horde of immune mechanisms that decide the fate of both the host as well as the pathogen. Our data may provide novel insights into the different molecular pathways of host response to virulent MTB infection, thereby stimulating new thoughts on the prevention of clinical tuberculous bacilli.

## Figures and Tables

**Table 1:** Number of differentially expressed proteins in macrophage-like cells after infection with different strains of *M. tuberculosis* at four tandem time points. U/D indicates the ratio of up-regulated proteins to down-regulated proteins.

	H37Ra			H37Rv			BND433			JAL2287		
Time	Up	Down	U/D	Up	Down	U/D	Up	Down	U/D	Up	Down	U/D
6 h	113	110	1.0	25	104	0.2	55	99	0.5	50	292	0.2
18 h	60	88	0.7	101	235	0.4	95	137	0.7	146	100	1.5
30 h	113	155	0.7	78	144	0.5	86	118	0.7	156	184	0.8
42 h	72	287	0.2	114	394	0.3	112	408	0.3	83	337	0.2

## Data Availability

The mass spectrometry proteomics data have been deposited in the ProteomeXchange Consortium via the PRIDE repository with the dataset identifier PXD022352 (https://www.ebi.ac.uk/pride/archive/projects/PXD022352).

## Supplementary Materials

The following supporting information can be downloaded at: https://www.mdpi.com/article/10.3390/microorganisms11122998/s1. Figure S1. Dynamic range of the protein abundance of macrophage-like cells upon infection with different strains of *M. tuberculosis*. XIC-based SWATH quantification values for the 1304 proteins were plotted with log10 intensity on the *y*-axis, and protein abundance rank on the *x*-axis. The plot shows a dynamic range of 4 orders of magnitude. Data shown were obtained from one typical set of experiments. Figure S2. Label-free DIA-SWATH analysis of macrophage-like cell proteome after infection with different strains of *M. tuberculosis* at 6 h of infection. Host cells were harvested and subjected to LC-MS/MS analysis following protein extraction and trypsin digestion. Left panel: The number of differentially expressed proteins detected in each dataset. The differentially expressed proteins were grouped into common virulent proteins, if they were shared between any of the three virulent strains (H37Rv, BND433, and JAL2287), and unique unshared proteins, if they were not shared among them. Right panel: Unsupervised hierarchical clustering and principal component analysis of the protein profiling of THP1 cells. Volcano plots with up- and down-regulated proteins (up/down ratio in parentheses) for each dataset are also shown. Con: control; Ra: H37Ra; Rv: H37Rv; BND: BND433; JAL: JAL2287. Figure S3. Biological processes were predicted for the differentially expressed proteins of macrophage-like cells after infection with different strains of *M. tuberculosis* at 6 h of infection. The differentially expressed proteins were grouped into common virulent proteins, if they were shared between any of the three virulent strains after infection (H37Rv, BND433, and JAL2287), and unique unshared proteins, if they were not shared among them. Left panel: up-regulated proteins; right panel: down-regulated proteins. Figure S4. Cellular components, molecular functions, and KEGG pathways were predicted for the common differentially expressed proteins of the host that were shared between any of the three virulent strains (H37Rv, BND433, and JAL2287) after infection at 6 h of incubation. Upper left panel: up-regulated proteins; upper right panel: down-regulated proteins. Lower panel: association networks of protein interactions with Markov clustering in circles were also constructed. Nodes in the diagram represent proteins and edges represent protein–protein associations. Figure S5. Label-free DIA-SWATH analysis of macrophage-like cell proteome after infection with different strains of *M. tuberculosis* at 30 h of infection. Host cells were harvested and subjected to LC-MS/MS analysis following protein extraction and trypsin digestion. Left panel: The number of differentially expressed proteins detected in each dataset. The differentially expressed proteins were grouped into common virulent proteins, if they were shared between any of the three virulent strains (H37Rv, BND433, and JAL2287), and unique unshared proteins, if they were not shared among them. Right panel: Unsupervised hierarchical clustering and principal component analysis of the protein profiling of THP1 cells. Volcano plots with up- and down-regulated proteins (up/down ratio in parentheses) for each dataset are also shown. Con: control; Ra: H37Ra; Rv: H37Rv; BND: BND433; JAL: JAL2287. Figure S6. Label-free DIA-SWATH analysis of macrophage-like cell proteome after infection with different strains of *M. tuberculosis* at 42 h of infection. Host cells were harvested and subjected to LC-MS/MS analysis following protein extraction and trypsin digestion. Left panel: The number of differentially expressed proteins detected in each dataset. The differentially expressed proteins were grouped into common virulent proteins, if they were shared between any of the three virulent strains (H37Rv, BND433, and JAL2287), and unique unshared proteins, if they were not shared among them. Right panel: Unsupervised hierarchical clustering and principal component analysis of the protein profiling of THP1 cells. Volcano plots with up- and down-regulated proteins (up/down ratio in parentheses) for each dataset are also shown. Con: control; Ra: H37Ra; Rv: H37Rv; BND: BND433; JAL: JAL2287. Figure S7. Biological processes were predicted for the differentially expressed proteins of macrophage-like cells after infection with different strains of *M. tuberculosis* at 30 h of infection. The differentially expressed proteins were grouped into common virulent proteins, if they were shared between any of the three virulent strain infections (H37Rv, BND433, and JAL2287), and unique unshared proteins, if they were not shared among them. Left panel: up-regulated proteins; right panel: down-regulated proteins. Figure S8. Cellular components, molecular functions, and KEGG pathways were predicted for the common differentially expressed proteins of the host that were shared between any of the three virulent strains (H37Rv, BND433, and JAL2287) after infection at 30 h of incubation. Upper left panel: up-regulated proteins; upper right panel: down-regulated proteins. Lower panel: association networks of protein interactions with Markov clustering in circles were also constructed. Nodes in the diagram represent proteins and edges represent protein–protein associations. Figure S9. Biological processes were predicted for the differentially expressed proteins of macrophage-like cells after infection with different strains of *M. tuberculosis* at 42 h of infection. The differentially expressed proteins were grouped into common virulent proteins, if they were shared between any of the three virulent strains after infection (H37Rv, BND433, and JAL2287), and unique unshared proteins, if they were not shared among them. Left panel: up-regulated proteins; right panel: down-regulated proteins. Figure S10. Cellular components, molecular functions, and KEGG pathways were predicted for the common differentially expressed proteins of the host that were shared between any of the three virulent strains (H37Rv, BND433, and JAL2287) after infection at 42 h of incubation. Upper left panel: up-regulated proteins; upper right panel: down-regulated proteins. Lower panel: association networks of protein interactions with Markov clustering in circles were also constructed. Nodes in the diagram represent proteins and edges represent protein–protein associations. Figure S11. Common differentially expressed proteins of the host that were shared between any of the three virulent strains (H37Rv, BND433, and JAL2287) at four different time points of infection. Table S1. Number of proteins quantified in macrophage-like cells after infection with different strains of *M. tuberculosis* at four tandem time points using label-free SWATH-MS. Infections of the host were carried out with MTB and cells were harvested at 6, 18, 30, and 42 h for proteomic analysis. UI: uninfected control; Ra: H37Ra infection; Rv: H37Rv infection; BND: BND433 infection; JAL: JAL2287 infection. Table S2. List of differentially expressed proteins in macrophage-like cells at 6 h post-infection with different strains of *M. tuberculosis*. Down-regulated proteins are shown in red. Table S3. List of differentially expressed proteins in macrophage-like cells at 18 h post-infection with different strains of *M. tuberculosis*. Down-regulated proteins are shown in red. Table S4. List of differentially expressed proteins in macrophage-like cells at 30 h post-infection with different strains of *M. tuberculosis*. Down-regulated proteins are shown in red. Table S5. List of differentially expressed proteins in macrophage-like cells at 42 h post-infection with different strains of *M. tuberculosis*. Down-regulated proteins are shown in red. Table S6. List of differentially expressed proteins in macrophage-like cells that shared at least two out of three virulent strain infections (H37Rv, BND433, and JAL2287) at different time points.
