# Supplementary material for: Temporal Profiling of Host Proteome against Different M. tuberculosis Strains Reveals Delayed Epigenetic Orchestration
Source: Microorganisms. 2023 Dec 16;11(12):2998. doi: 10.3390/microorganisms11122998 (PMC10745383; doi:10.3390/microorganisms11122998)
Supplement: Supplementary file 1 [file microorganisms-11-02998-s001.zip › Supplementary Figures.pdf]

Figure S1

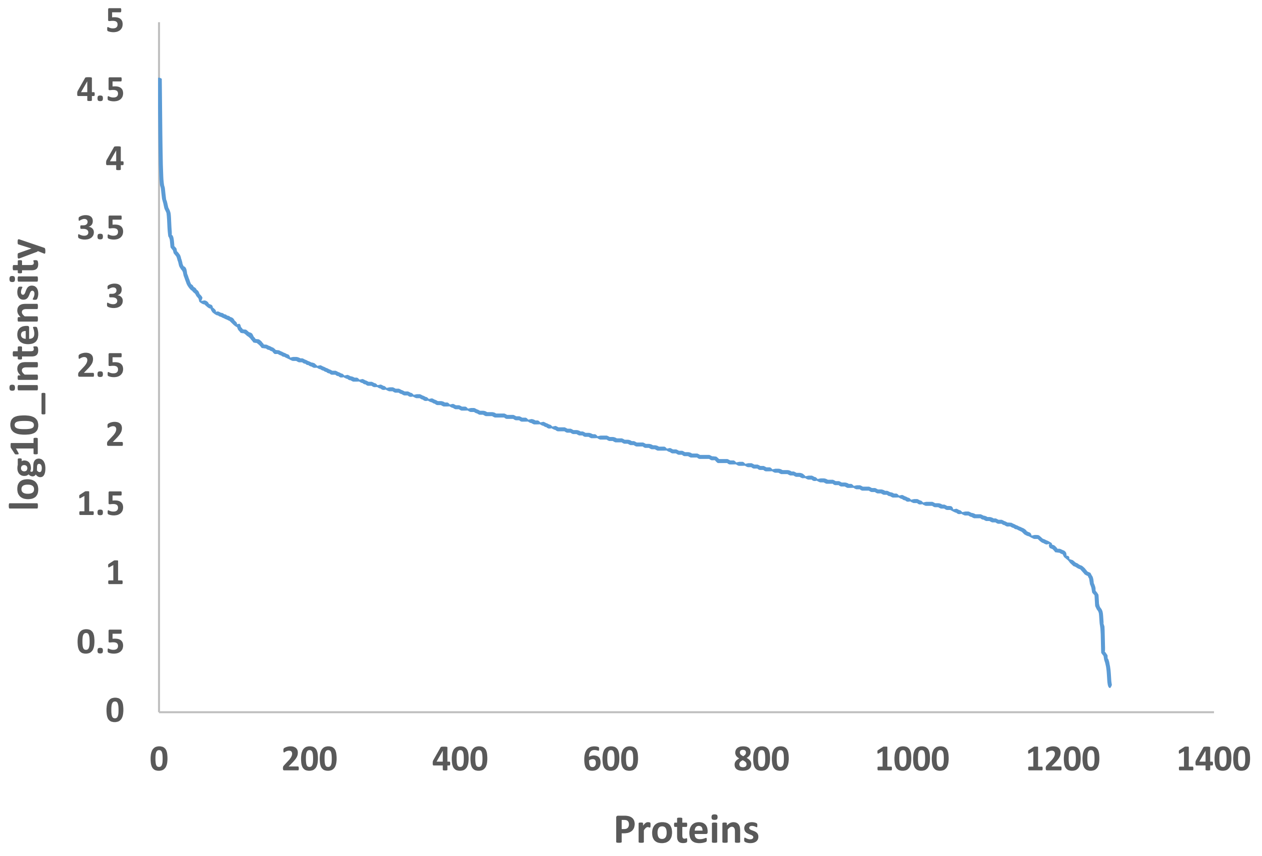

5
4.5
4
3.5
3
2.5
2
1.5
1
0.5
0
log10_intensity
0
200
400
600
800
1000
1200
1400
Proteins

Figure S2

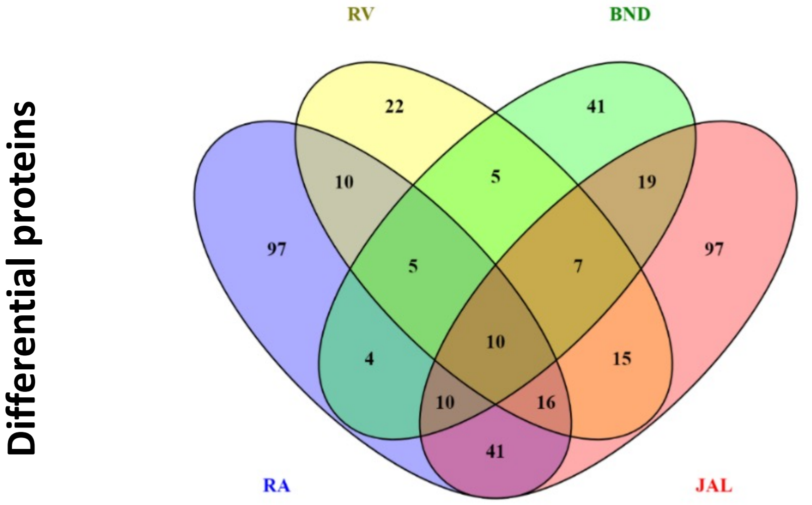

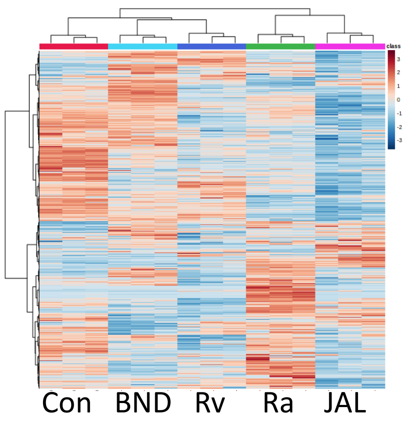

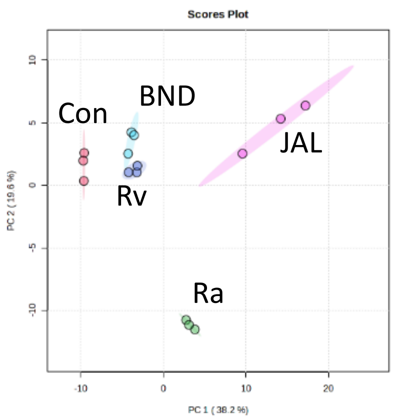

Common virulent				Unique unshared			
Rv-BND	Rv-JAL	BND-JAL	Rv-BND-JAL	Ra	Rv	BND	JAL
5	15	19	7	97	22	41	97

Ra - up: 113, down: 110 (1.0)

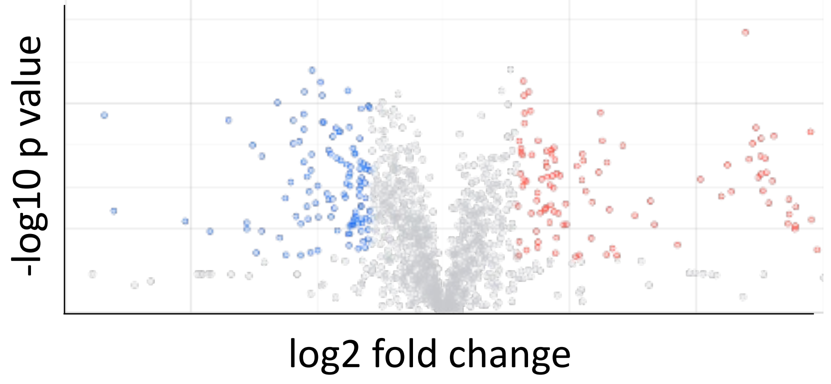

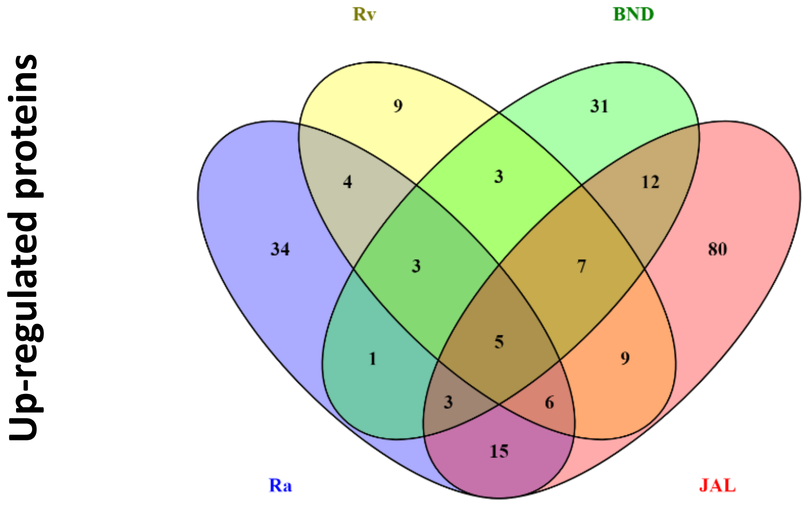

Rv - up: 25, down: 104 (0.2)

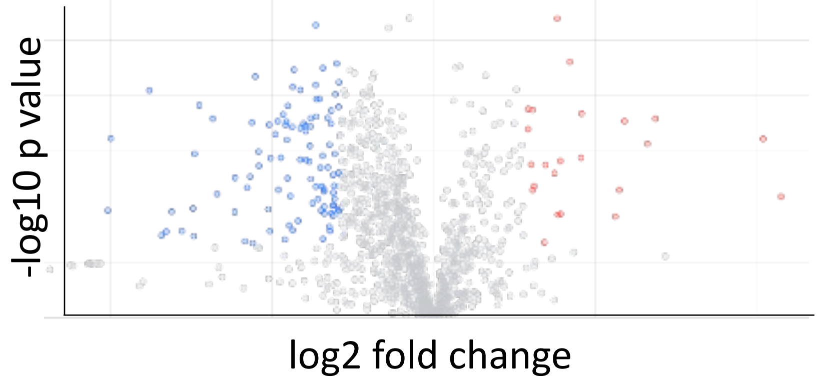

Common virulent				Unique unshared			
Rv-BND	Rv-JAL	BND-JAL	Rv-BND-JAL	Ra	Rv	BND	JAL
3	9	12	7	34	9	31	80

BND - up: 55, down: 99 (0.5)

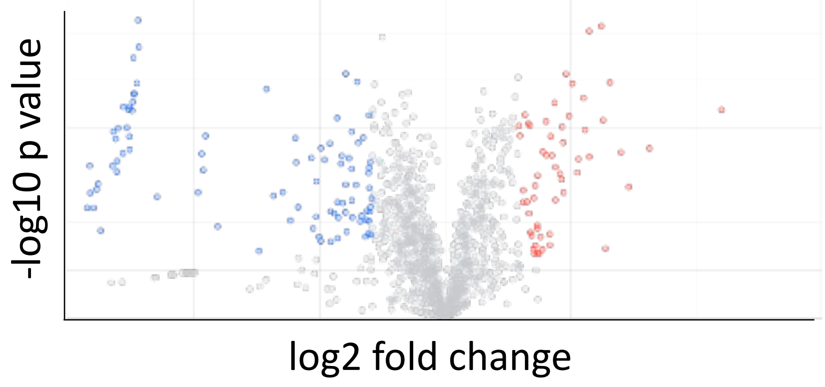

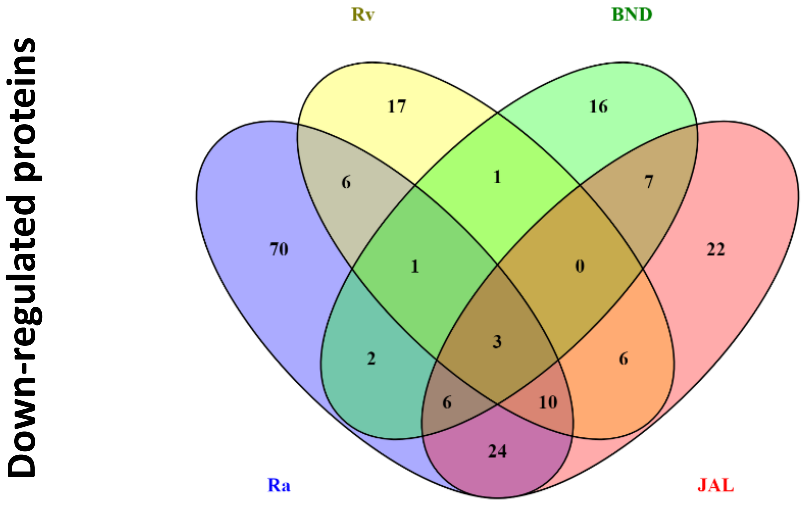

JAL - up: 50, down: 292 (0.2)

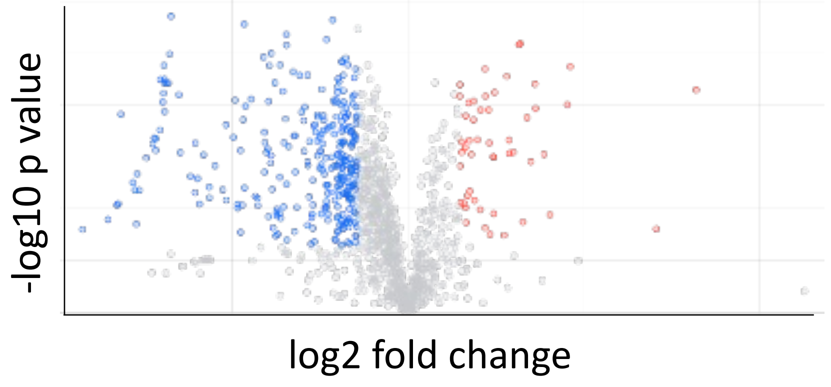

Common virulent				Unique unshared			
Rv-BND	Rv-JAL	BND-JAL	Rv-BND-JAL	Ra	Rv	BND	JAL
1	6	7	0	70	17	16	22

Figure S3

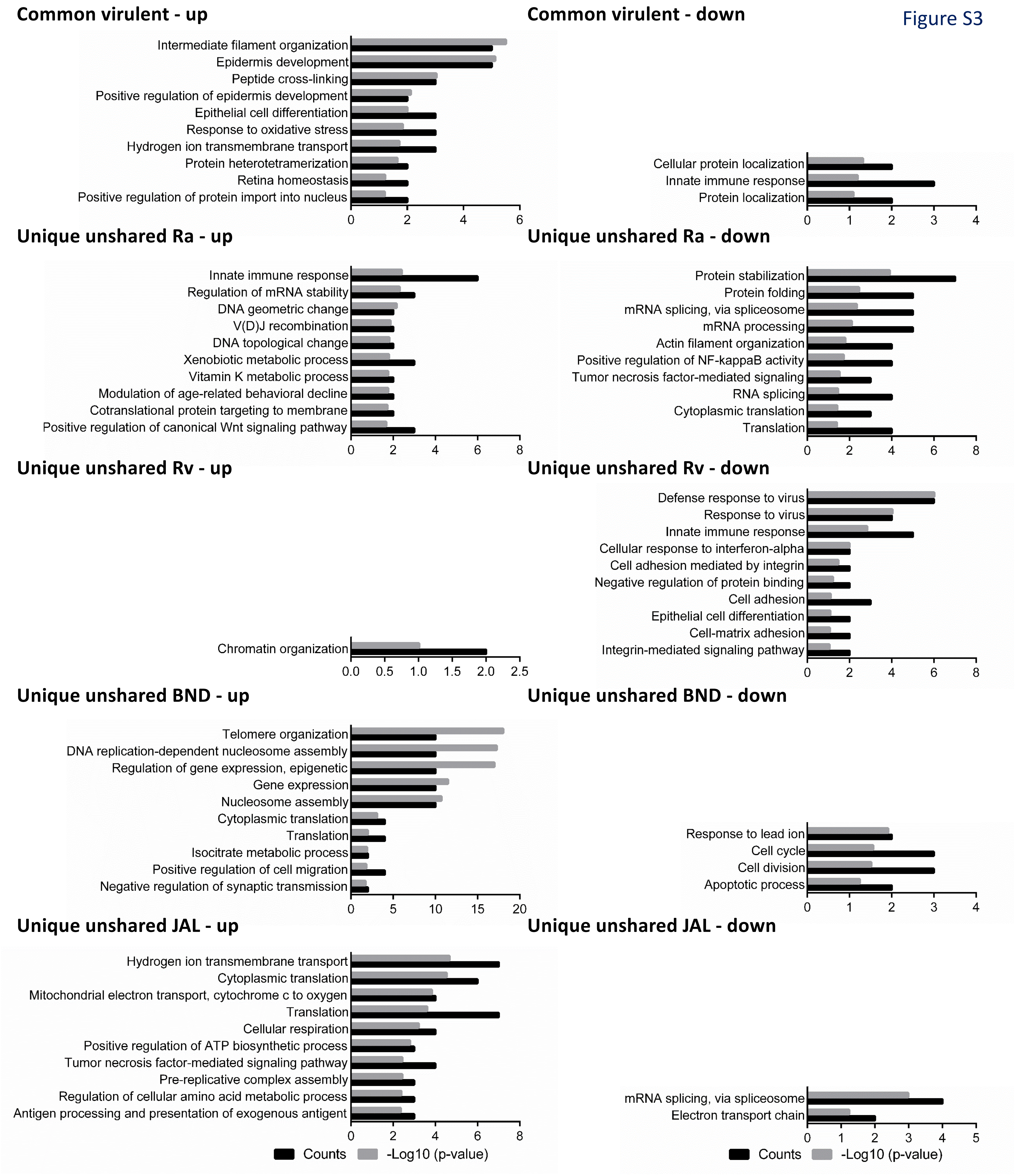
Common virulent - up
Intermediate filament organization
Epidermis development
Peptide cross-linking
Positive regulation of epidermis development
Epithelial cell differentiation
Response to oxidative stress
Hydrogen ion transmembrane transport
Protein heterotetramerization
Retina homeostasis
Positive regulation of protein import into nucleus
0 2 4 6
Common virulent - down
Cellular protein localization
Innate immune response
Protein localization
0 1 2 3 4
Unique unshared Ra - up
Innate immune response
Regulation of mRNA stability
DNA geometric change
V(D)J recombination
DNA topological change
Xenobiotic metabolic process
Vitamin K metabolic process
Modulation of age-related behavioral decline
Cotranslational protein targeting to membrane
Positive regulation of canonical Wnt signaling pathway
0 2 4 6 8
Unique unshared Ra - down
Protein stabilization
Protein folding
mRNA splicing, via spliceosome
mRNA processing
Actin filament organization
Positive regulation of NF-kappaB activity
Tumor necrosis factor-mediated signaling
RNA splicing
Cytoplasmic translation
Translation
0 2 4 6 8
Unique unshared Rv - up
Chromatin organization
0.0 0.5 1.0 1.5 2.0 2.5
Unique unshared Rv - down
Defense response to virus
Response to virus
Innate immune response
Cellular response to interferon-alpha
Cell adhesion mediated by integrin
Negative regulation of protein binding
Cell adhesion
Epithelial cell differentiation
Cell-matrix adhesion
Integrin-mediated signaling pathway
0 2 4 6 8
Unique unshared BND - up
Telomere organization
DNA replication-dependent nucleosome assembly
Regulation of gene expression, epigenetic
Gene expression
Nucleosome assembly
Cytoplasmic translation
Translation
Isocitrate metabolic process
Positive regulation of cell migration
Negative regulation of synaptic transmission
0 5 10 15 20
Unique unshared BND - down
Response to lead ion
Cell cycle
Cell division
Apoptotic process
0 1 2 3 4
Unique unshared JAL - up
Hydrogen ion transmembrane transport
Cytoplasmic translation
Mitochondrial electron transport, cytochrome c to oxygen
Translation
Cellular respiration
Positive regulation of ATP biosynthetic process
Tumor necrosis factor-mediated signaling pathway
Pre-replicative complex assembly
Regulation of cellular amino acid metabolic process
Antigen processing and presentation of exogenous antigent
0 2 4 6 8
Counts -Log10 (p-value)
Unique unshared JAL - down
mRNA splicing, via spliceosome
Electron transport chain
0 1 2 3 4 5
Counts -Log10 (p-value)

Figure S4

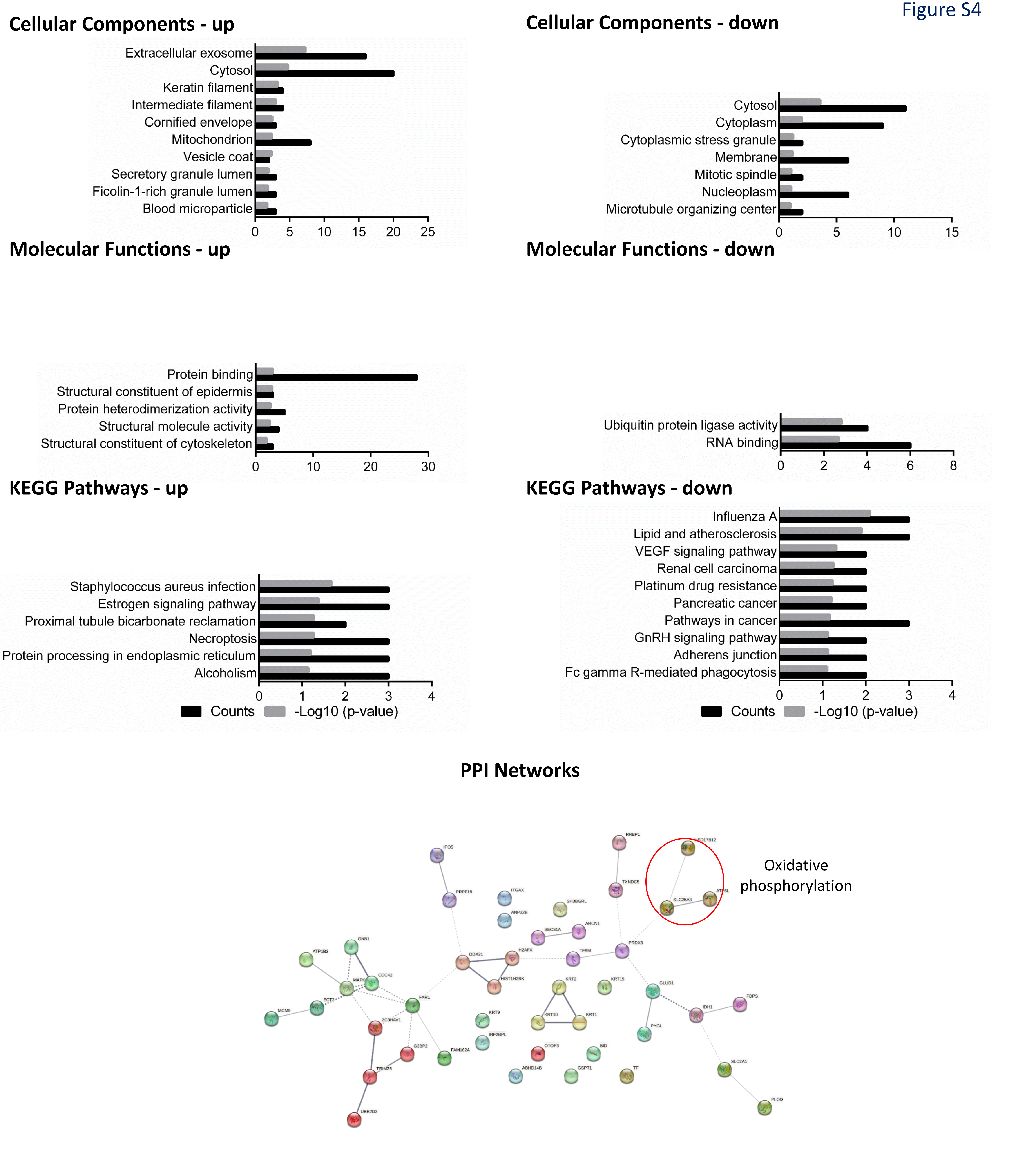

Cellular Components - up
Extracellular exosome
Cytosol
Keratin filament
Intermediate filament
Cornified envelope
Mitochondrion
Vesicle coat
Secretory granule lumen
Ficolin-1-rich granule lumen
Blood microparticle
0 5 10 15 20 25
Cellular Components - down
Cytosol
Cytoplasm
Cytoplasmic stress granule
Membrane
Mitotic spindle
Nucleoplasm
Microtubule organizing center
0 5 10 15
Molecular Functions - up
Protein binding
Structural constituent of epidermis
Protein heterodimerization activity
Structural molecule activity
Structural constituent of cytoskeleton
0 10 20 30
Molecular Functions - down
Ubiquitin protein ligase activity
RNA binding
0 2 4 6 8
KEGG Pathways - up
Staphylococcus aureus infection
Estrogen signaling pathway
Proximal tubule bicarbonate reclamation
Necroptosis
Protein processing in endoplasmic reticulum
Alcoholism
0 1 2 3 4
Counts -Log10 (p-value)
KEGG Pathways - down
Influenza A
Lipid and atherosclerosis
VEGF signaling pathway
Renal cell carcinoma
Platinum drug resistance
Pancreatic cancer
Pathways in cancer
GnRH signaling pathway
Adherens junction
Fc gamma R-mediated phagocytosis
0 1 2 3 4
Counts -Log10 (p-value)
PPI Networks
Oxidative phosphorylation

Figure S5

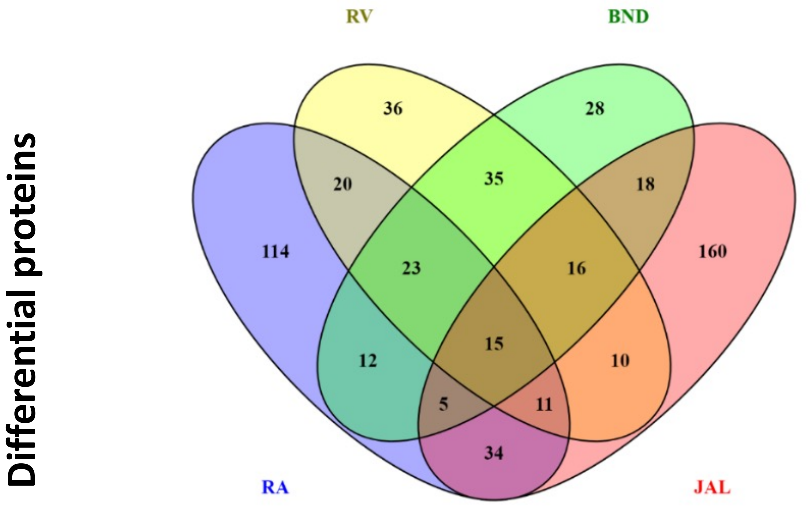

Common virulent				Unique unshared			
Rv-BND	Rv-JAL	BND-JAL	Rv-BND-JAL	Ra	Rv	BND	JAL
35	10	18	16	114	36	28	160

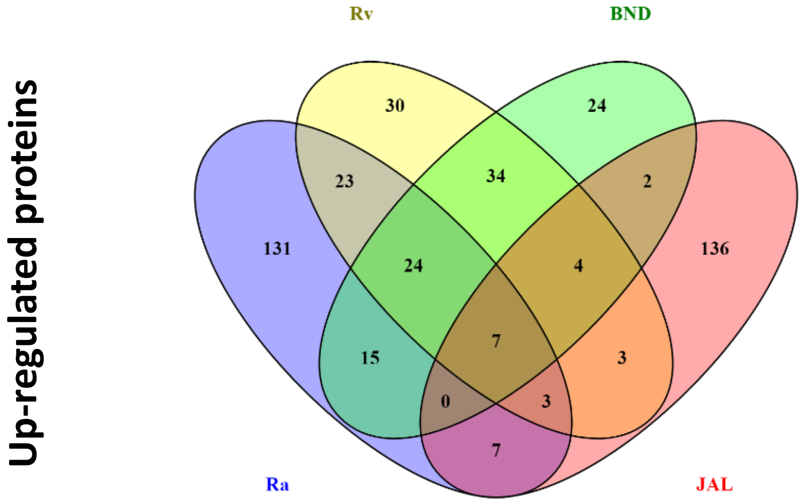

Common virulent				Unique unshared			
Rv-BND	Rv-JAL	BND-JAL	Rv-BND-JAL	Ra	Rv	BND	JAL
34	3	2	4	131	30	24	136

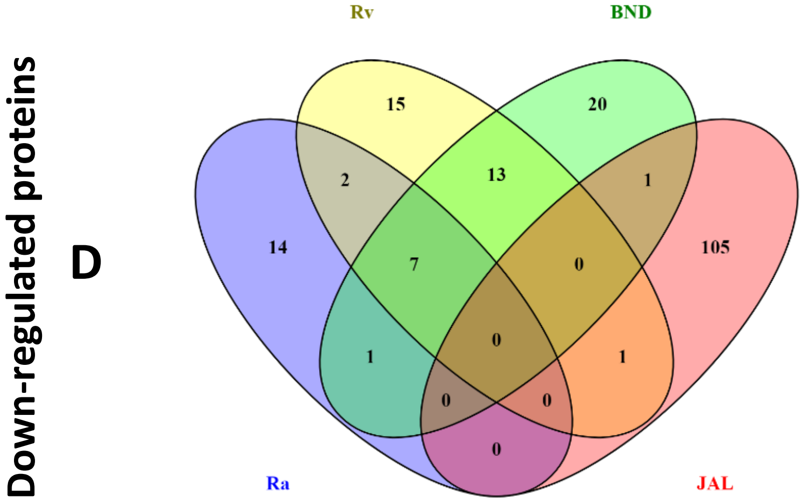

Common virulent				Unique unshared			
Rv-BND	Rv-JAL	BND-JAL	Rv-BND-JAL	Ra	Rv	BND	JAL
13	1	1	0	14	15	20	105

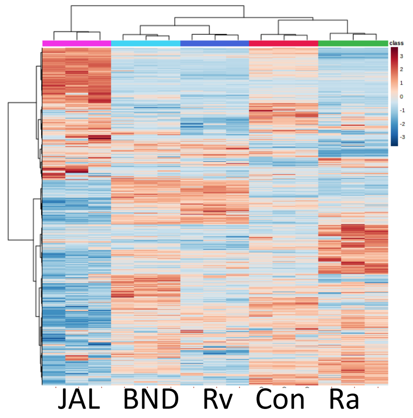

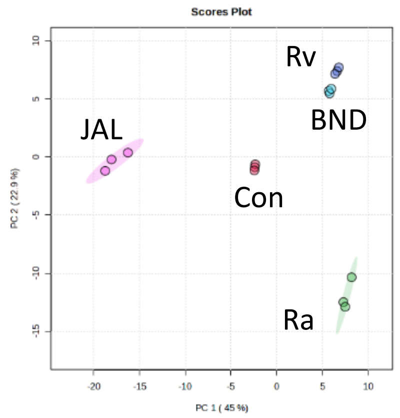

Ra - up: 113, down: 155 (0.7)

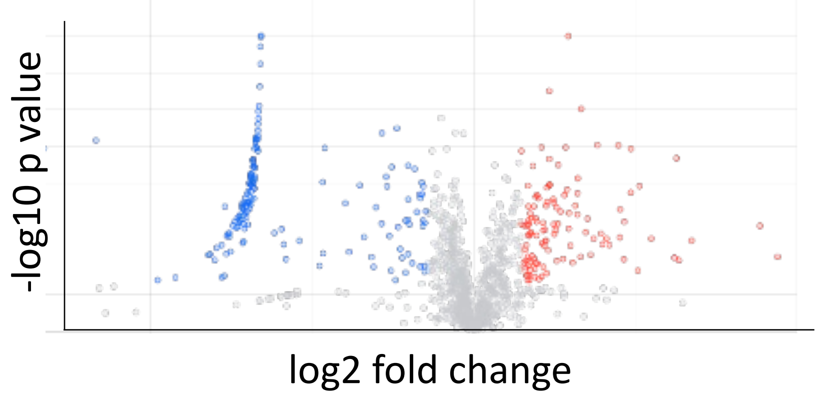

Rv - up: 78, down: 144 (0.5)

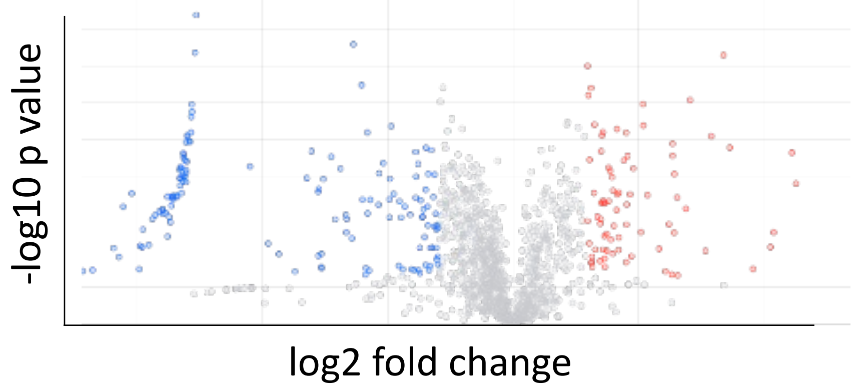

BND - up: 86, down: 118 (0.7)

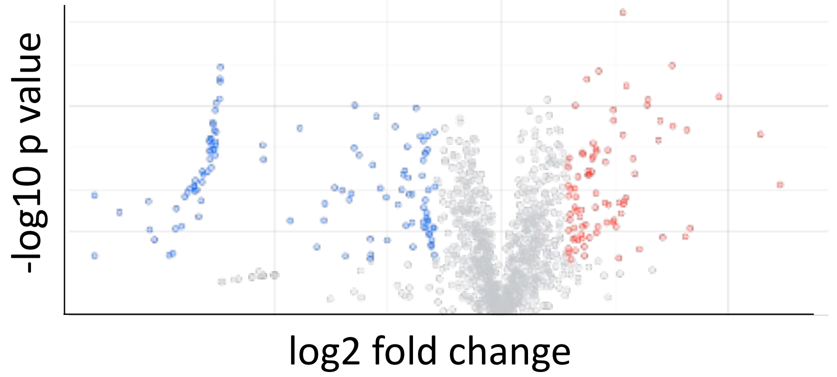

JAL - up: 156, down: 184 (0.8)

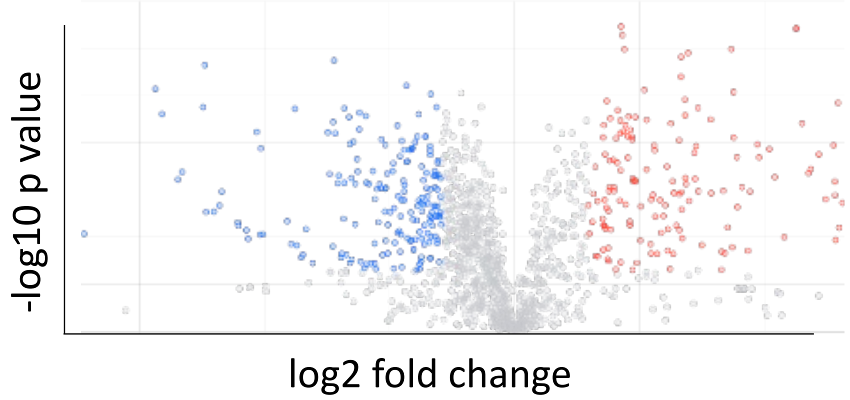

Figure S6

Differential proteins

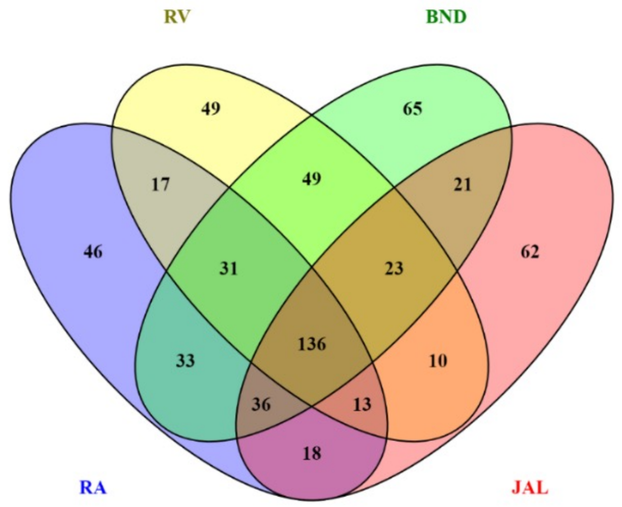

Common virulent				Unique unshared			
Rv-BND	Rv-JAL	BND-JAL	Rv-BND-JAL	Ra	Rv	BND	JAL
49	10	21	23	46	49	65	62

Up-regulated proteins

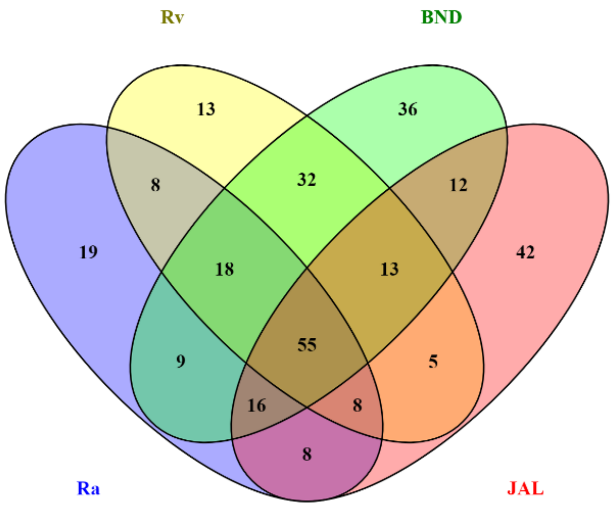

Common virulent				Unique unshared			
Rv-BND	Rv-JAL	BND-JAL	Rv-BND-JAL	Ra	Rv	BND	JAL
32	5	12	13	19	13	36	42

Down-regulated proteins

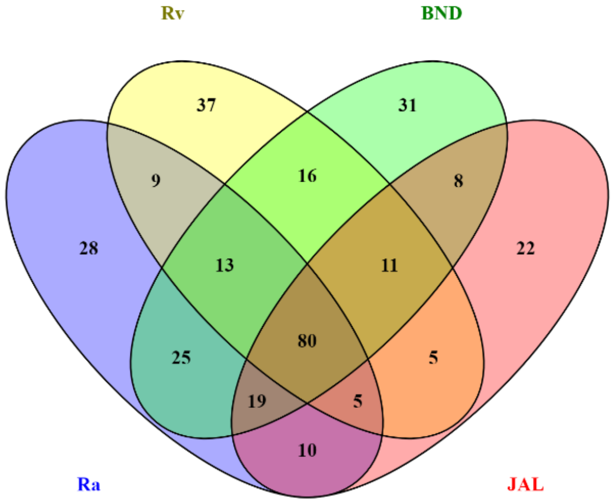

Common virulent				Unique unshared			
Rv-BND	Rv-JAL	BND-JAL	Rv-BND-JAL	Ra	Rv	BND	JAL
16	5	8	11	28	37	31	22

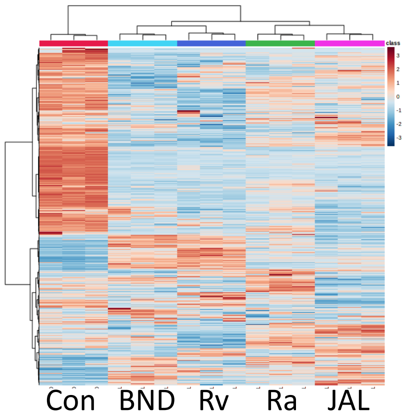

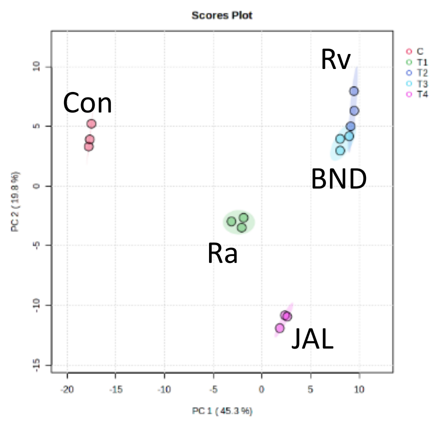

Ra - up: 72, down: 287 (0.2)

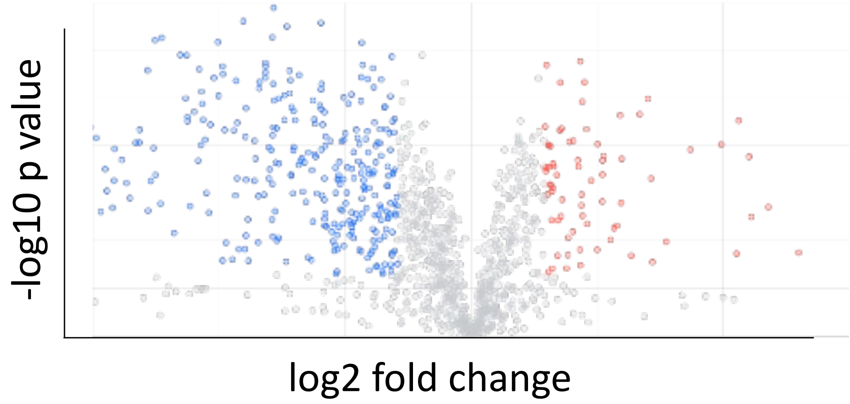

Rv - up: 114, down: 394 (0.3)

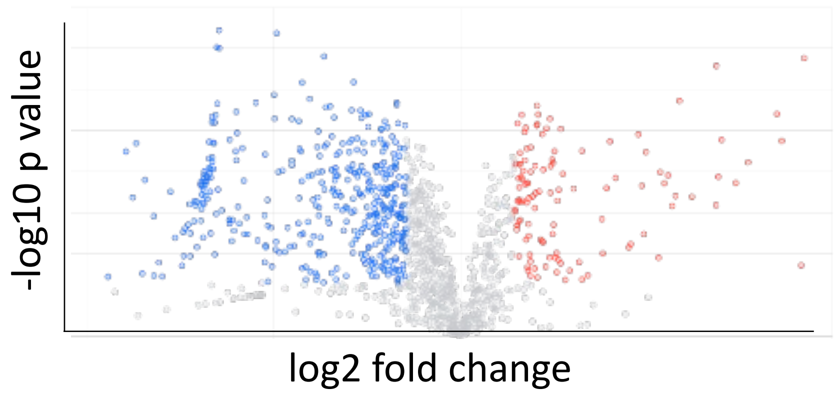

BND - up: 112, down: 408 (0.3)

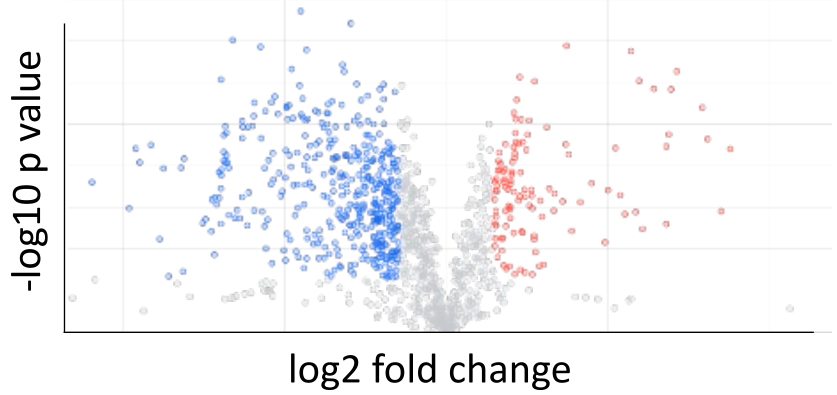

JAL - up: 83, down: 337 (0.2)

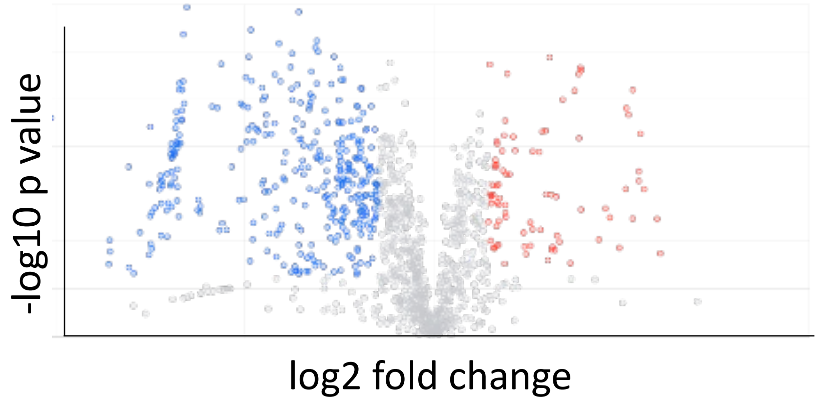

Figure S7

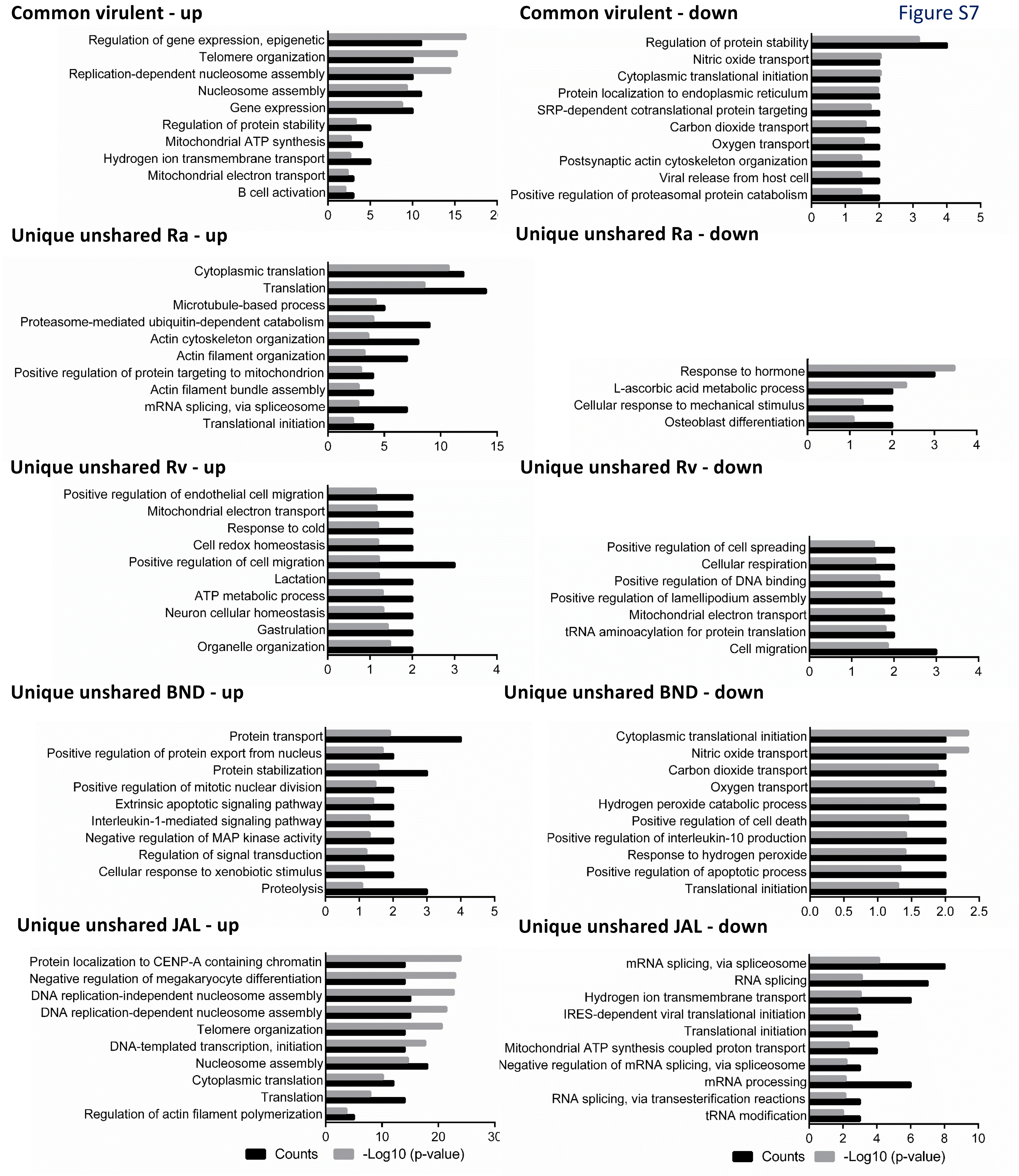

Common virulent - up
Regulation of gene expression, epigenetic
Telomere organization
Replication-dependent nucleosome assembly
Nucleosome assembly
Gene expression
Regulation of protein stability
Mitochondrial ATP synthesis
Hydrogen ion transmembrane transport
Mitochondrial electron transport
B cell activation
0 5 10 15 2
Common virulent - down
Regulation of protein stability
Nitric oxide transport
Cytoplasmic translational initiation
Protein localization to endoplasmic reticulum
SRP-dependent cotranslational protein targeting
Carbon dioxide transport
Oxygen transport
Postsynaptic actin cytoskeleton organization
Viral release from host cell
Positive regulation of proteasomal protein catabolism
0 1 2 3 4 5
Unique unshared Ra - up
Cytoplasmic translation
Translation
Microtubule-based process
Proteasome-mediated ubiquitin-dependent catabolism
Actin cytoskeleton organization
Actin filament organization
Positive regulation of protein targeting to mitochondrion
Actin filament bundle assembly
mRNA splicing, via spliceosome
Translational initiation
0 5 10 15
Unique unshared Ra - down
Response to hormone
L-ascorbic acid metabolic process
Cellular response to mechanical stimulus
Osteoblast differentiation
0 1 2 3 4
Unique unshared Rv - up
Positive regulation of endothelial cell migration
Mitochondrial electron transport
Response to cold
Cell redox homeostasis
Positive regulation of cell migration
Lactation
ATP metabolic process
Neuron cellular homeostasis
Gastrulation
Organelle organization
0 1 2 3 4
Unique unshared Rv - down
Positive regulation of cell spreading
Cellular respiration
Positive regulation of DNA binding
Positive regulation of lamellipodium assembly
Mitochondrial electron transport
tRNA aminoacylation for protein translation
Cell migration
0 1 2 3 4
Unique unshared BND - up
Protein transport
Positive regulation of protein export from nucleus
Protein stabilization
Positive regulation of mitotic nuclear division
Extrinsic apoptotic signaling pathway
Interleukin-1-mediated signaling pathway
Negative regulation of MAP kinase activity
Regulation of signal transduction
Cellular response to xenobiotic stimulus
Proteolysis
0 1 2 3 4 5
Unique unshared BND - down
Cytoplasmic translational initiation
Nitric oxide transport
Carbon dioxide transport
Oxygen transport
Hydrogen peroxide catabolic process
Positive regulation of cell death
Positive regulation of interleukin-10 production
Response to hydrogen peroxide
Positive regulation of apoptotic process
Translational initiation
0.0 0.5 1.0 1.5 2.0 2.5
Unique unshared JAL - up
Protein localization to CENP-A containing chromatin
Negative regulation of megakaryocyte differentiation
DNA replication-independent nucleosome assembly
DNA replication-dependent nucleosome assembly
Telomere organization
DNA-templated transcription, initiation
Nucleosome assembly
Cytoplasmic translation
Translation
Regulation of actin filament polymerization
0 10 20 30
Counts -Log10 (p-value)
Unique unshared JAL - down
mRNA splicing, via spliceosome
RNA splicing
Hydrogen ion transmembrane transport
IRES-dependent viral translational initiation
Translational initiation
Mitochondrial ATP synthesis coupled proton transport
Negative regulation of mRNA splicing, via spliceosome
mRNA processing
RNA splicing, via transesterification reactions
tRNA modification
0 2 4 6 8 10
Counts -Log10 (p-value)

Figure S8

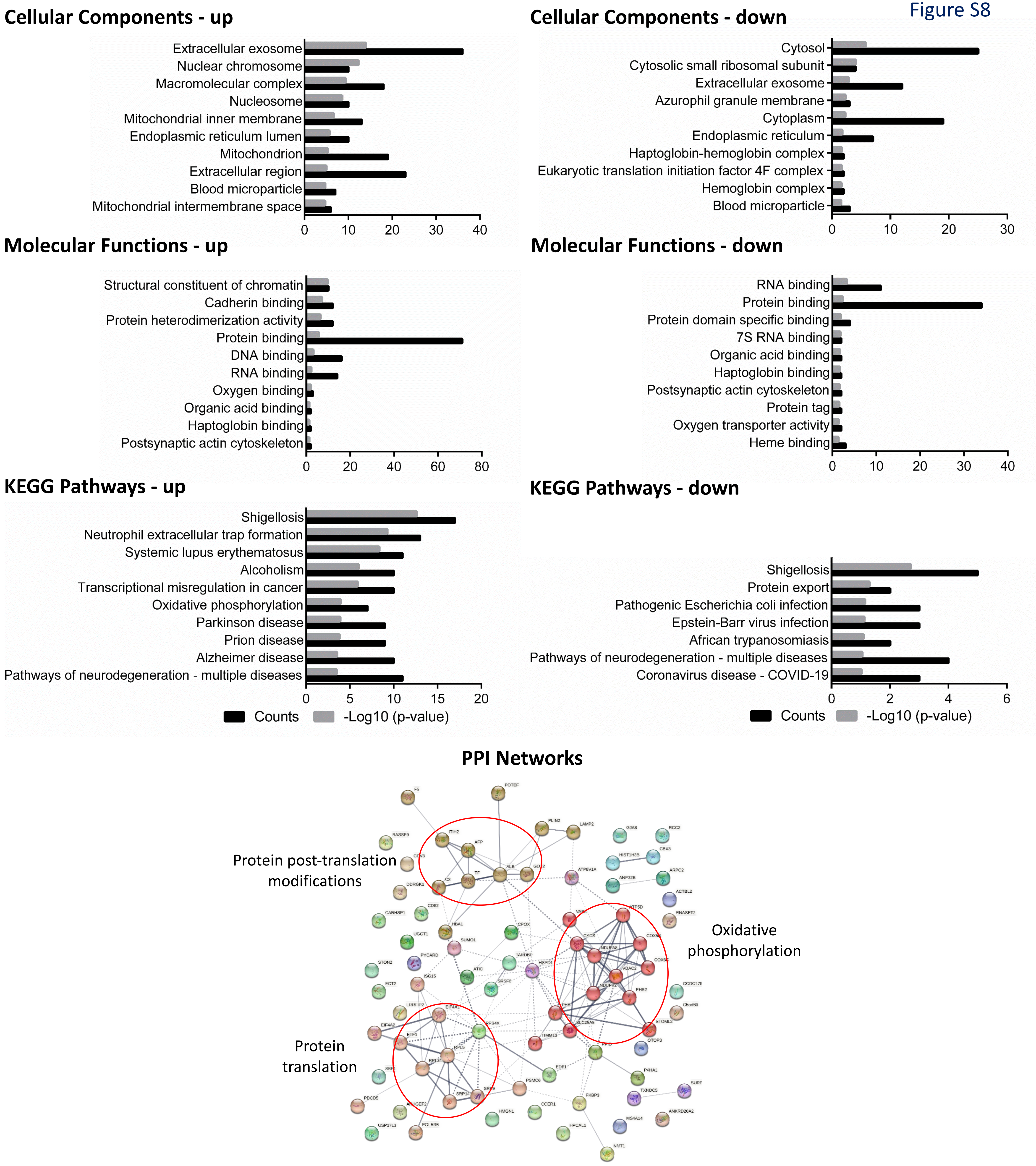
Cellular Components - up
Extracellular exosome
Nuclear chromosome
Macromolecular complex
Nucleosome
Mitochondrial inner membrane
Endoplasmic reticulum lumen
Mitochondrion
Extracellular region
Blood microparticle
Mitochondrial intermembrane space
0 10 20 30 40
Cellular Components - down
Cytosol
Cytosolic small ribosomal subunit
Extracellular exosome
Azurophil granule membrane
Cytoplasm
Endoplasmic reticulum
Haptoglobin-hemoglobin complex
Eukaryotic translation initiation factor 4F complex
Hemoglobin complex
Blood microparticle
0 10 20 30
Molecular Functions - up
Structural constituent of chromatin
Cadherin binding
Protein heterodimerization activity
Protein binding
DNA binding
RNA binding
Oxygen binding
Organic acid binding
Haptoglobin binding
Postsynaptic actin cytoskeleton
0 20 40 60 80
Molecular Functions - down
RNA binding
Protein binding
Protein domain specific binding
7S RNA binding
Organic acid binding
Haptoglobin binding
Postsynaptic actin cytoskeleton
Protein tag
Oxygen transporter activity
Heme binding
0 10 20 30 40
KEGG Pathways - up
Shigellosis
Neutrophil extracellular trap formation
Systemic lupus erythematosus
Alcoholism
Transcriptional misregulation in cancer
Oxidative phosphorylation
Parkinson disease
Prion disease
Alzheimer disease
Pathways of neurodegeneration - multiple diseases
0 5 10 15 20
Counts
-Log10 (p-value)
KEGG Pathways - down
Shigellosis
Protein export
Pathogenic Escherichia coli infection
Epstein-Barr virus infection
African trypanosomiasis
Pathways of neurodegeneration - multiple diseases
Coronavirus disease - COVID-19
0 2 4 6
Counts
-Log10 (p-value)
PPI Networks
Protein post-translation modifications
Oxidative phosphorylation
Protein translation

Figure S9

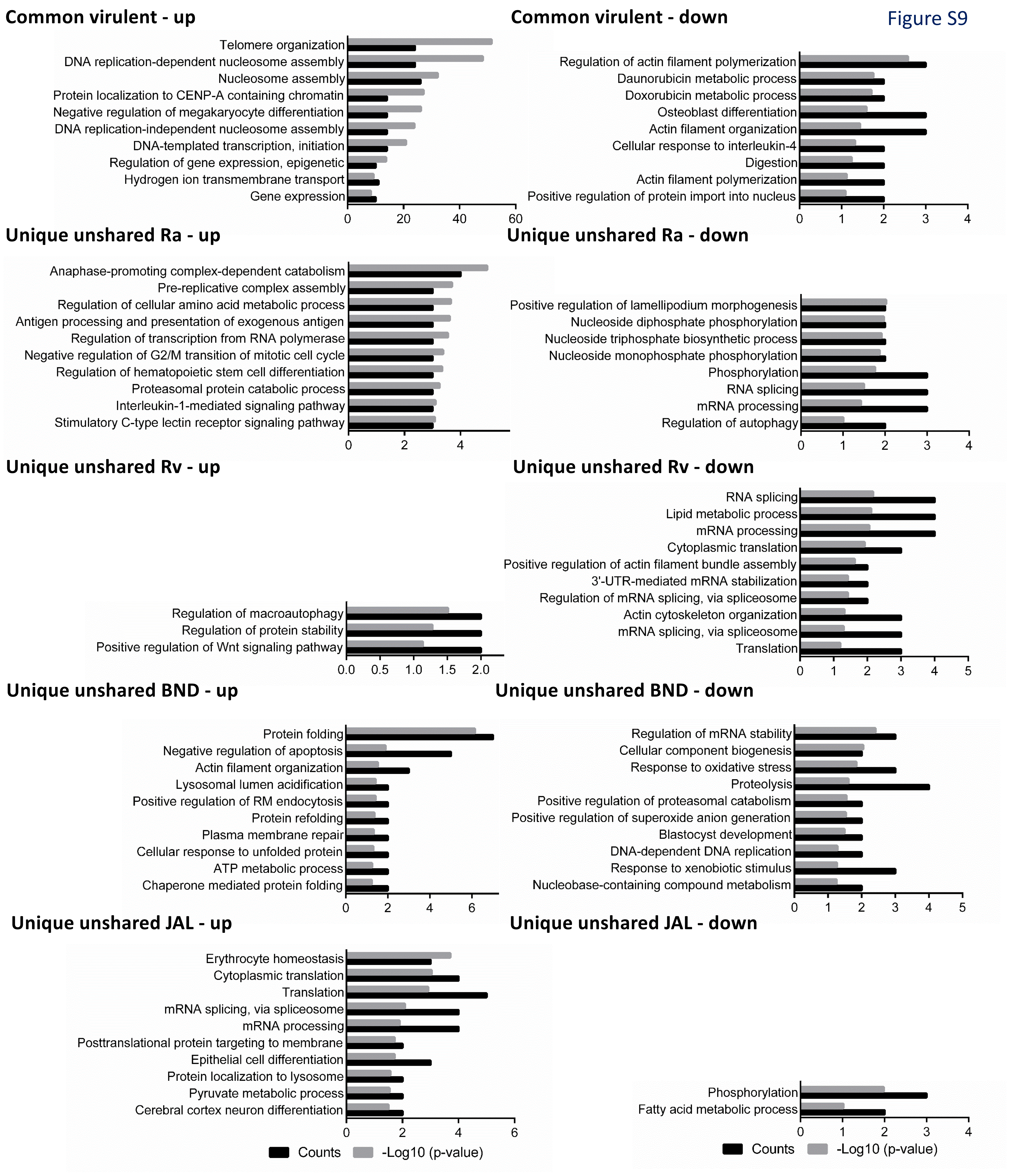

Common virulent - up
Telomere organization
DNA replication-dependent nucleosome assembly
Nucleosome assembly
Protein localization to CENP-A containing chromatin
Negative regulation of megakaryocyte differentiation
DNA replication-independent nucleosome assembly
DNA-templated transcription, initiation
Regulation of gene expression, epigenetic
Hydrogen ion transmembrane transport
Gene expression
0 20 40 60
Common virulent - down
Regulation of actin filament polymerization
Daunorubicin metabolic process
Doxorubicin metabolic process
Osteoblast differentiation
Actin filament organization
Cellular response to interleukin-4
Digestion
Actin filament polymerization
Positive regulation of protein import into nucleus
0 1 2 3 4
Unique unshared Ra - up
Anaphase-promoting complex-dependent catabolism
Pre-replicative complex assembly
Regulation of cellular amino acid metabolic process
Antigen processing and presentation of exogenous antigen
Regulation of transcription from RNA polymerase
Negative regulation of G2/M transition of mitotic cell cycle
Regulation of hematopoietic stem cell differentiation
Proteasomal protein catabolic process
Interleukin-1-mediated signaling pathway
Stimulatory C-type lectin receptor signaling pathway
0 2 4
Unique unshared Ra - down
Positive regulation of lamellipodium morphogenesis
Nucleoside diphosphate phosphorylation
Nucleoside triphosphate biosynthetic process
Nucleoside monophosphate phosphorylation
Phosphorylation
RNA splicing
mRNA processing
Regulation of autophagy
0 1 2 3 4
Unique unshared Rv - up
Regulation of macroautophagy
Regulation of protein stability
Positive regulation of Wnt signaling pathway
0.0 0.5 1.0 1.5 2.0
Unique unshared Rv - down
RNA splicing
Lipid metabolic process
mRNA processing
Cytoplasmic translation
Positive regulation of actin filament bundle assembly
3'-UTR-mediated mRNA stabilization
Regulation of mRNA splicing, via spliceosome
Actin cytoskeleton organization
mRNA splicing, via spliceosome
Translation
0 1 2 3 4 5
Unique unshared BND - up
Protein folding
Negative regulation of apoptosis
Actin filament organization
Lysosomal lumen acidification
Positive regulation of RM endocytosis
Protein refolding
Plasma membrane repair
Cellular response to unfolded protein
ATP metabolic process
Chaperone mediated protein folding
0 2 4 6
Unique unshared BND - down
Regulation of mRNA stability
Cellular component biogenesis
Response to oxidative stress
Proteolysis
Positive regulation of proteasomal catabolism
Positive regulation of superoxide anion generation
Blastocyst development
DNA-dependent DNA replication
Response to xenobiotic stimulus
Nucleobase-containing compound metabolism
0 1 2 3 4 5
Unique unshared JAL - up
Erythrocyte homeostasis
Cytoplasmic translation
Translation
mRNA splicing, via spliceosome
mRNA processing
Posttranslational protein targeting to membrane
Epithelial cell differentiation
Protein localization to lysosome
Pyruvate metabolic process
Cerebral cortex neuron differentiation
0 2 4 6
Counts -Log10 (p-value)
Unique unshared JAL - down
Phosphorylation
Fatty acid metabolic process
0 1 2 3 4
Counts -Log10 (p-value)

Figure S10

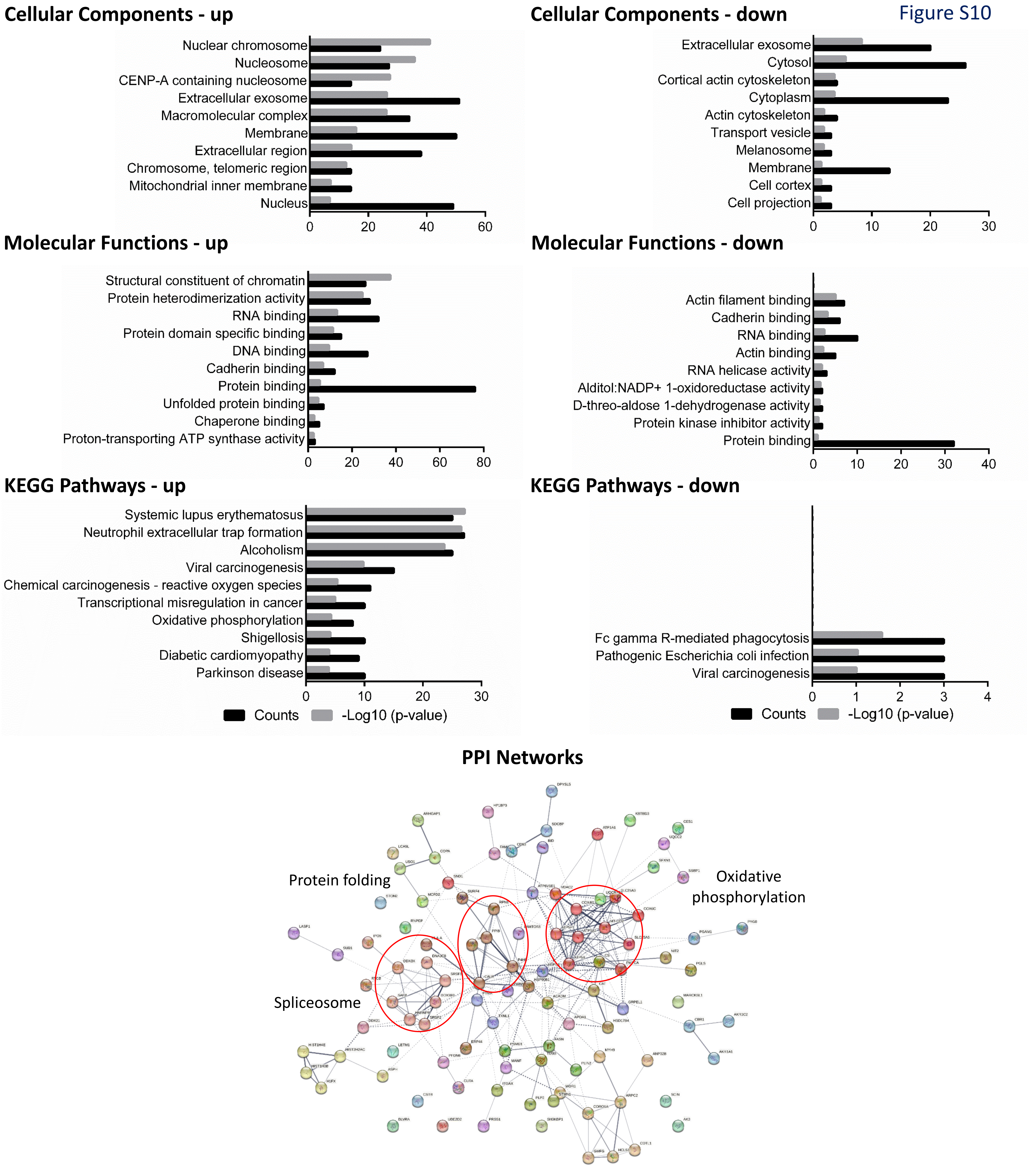
Cellular Components - up
Nuclear chromosome
Nucleosome
CENP-A containing nucleosome
Extracellular exosome
Macromolecular complex
Membrane
Extracellular region
Chromosome, telomeric region
Mitochondrial inner membrane
Nucleus
0 20 40 60
Cellular Components - down
Extracellular exosome
Cytosol
Cortical actin cytoskeleton
Cytoplasm
Actin cytoskeleton
Transport vesicle
Melanosome
Membrane
Cell cortex
Cell projection
0 10 20 30
Molecular Functions - up
Structural constituent of chromatin
Protein heterodimerization activity
RNA binding
Protein domain specific binding
DNA binding
Cadherin binding
Protein binding
Unfolded protein binding
Chaperone binding
Proton-transporting ATP synthase activity
0 20 40 60 80
Molecular Functions - down
Actin filament binding
Cadherin binding
RNA binding
Actin binding
RNA helicase activity
Alditol:NADP+ 1-oxidoreductase activity
D-threo-aldose 1-dehydrogenase activity
Protein kinase inhibitor activity
Protein binding
0 10 20 30 40
KEGG Pathways - up
Systemic lupus erythematosus
Neutrophil extracellular trap formation
Alcoholism
Viral carcinogenesis
Chemical carcinogenesis - reactive oxygen species
Transcriptional misregulation in cancer
Oxidative phosphorylation
Shigellosis
Diabetic cardiomyopathy
Parkinson disease
0 10 20 30
Counts -Log10 (p-value)
KEGG Pathways - down
Fc gamma R-mediated phagocytosis
Pathogenic Escherichia coli infection
Viral carcinogenesis
0 1 2 3 4
Counts -Log10 (p-value)
PPI Networks
Protein folding
Oxidative phosphorylation
Spliceosome

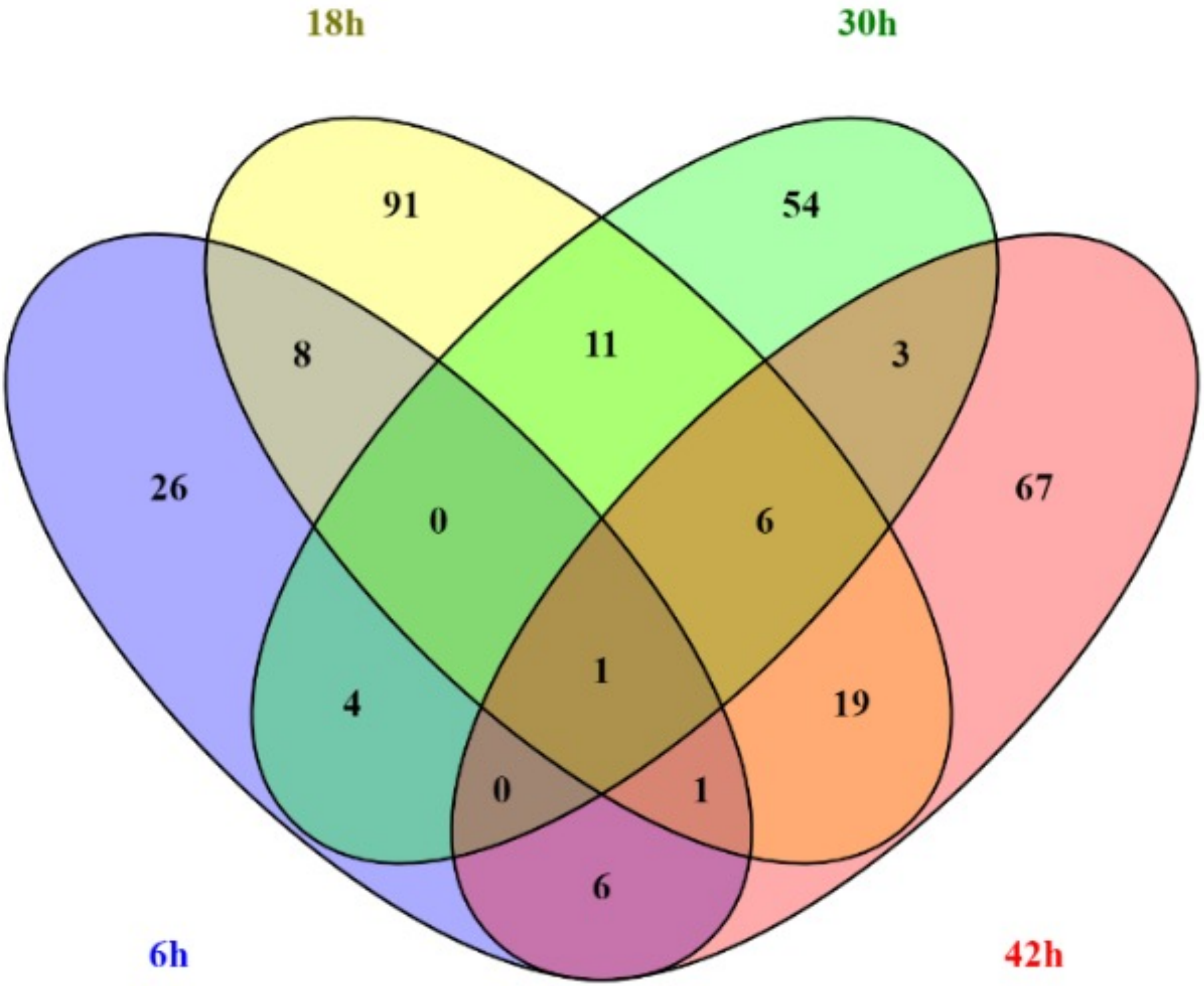
18h
30h
91
54
8
11
3
26
0
6
67
1
4
19
0
1
6
6h
42h
